# DC-SIGN–LEF1/TCF1–miR-185 feedback loop promotes colorectal cancer invasion and metastasis

**DOI:** 10.1038/s41418-019-0361-2

**Published:** 2019-06-19

**Authors:** Menglang Yuan, Xinsheng Zhang, Jingbo Zhang, Keyong Wang, Yu Zhang, Wei Shang, Yinan Zhang, Jingyi Cui, Xiaomeng Shi, Heya Na, Deyu Fang, Yunfei Zuo, Shuangyi Ren

**Affiliations:** 1 Department of General Surgery, The Second Affiliated Hospital of Dalian Medical University, 116023 Dalian, China; 2 Department of Clinical Biochemistry, College of Laboratory Diagnostic Medicine, Dalian Medical University, 116044 Dalian, China; 3 Department of Pathology, Northwestern University Feinberg School of Medicine, Chicago, IL 60611 USA

**Keywords:** Metastasis, Oncogenes

## Abstract

DC-SIGN is previously focused on its physiologic and pathophysiologic roles in immune cells. Little is known about whether DC-SIGN is expressed in malignant epithelial cells and how DC-SIGN participates in tumor progression. Here we showed that DC-SIGN expression was increased in metastatic colorectal cancer (CRC) cell lines and patient tissues. The overall survival in CRC patients with positive DC-SIGN was remarkably reduced. Gain of DC-SIGN function facilitated the CRC metastases both in vitro and in vivo, and this effect was reversed by miR-185. DC-SIGN and Lyn interacted physically, and Lyn maintained the stability of DC-SIGN in cells. DC-SIGN activation recruited Lyn and p85 to form the DC-SIGN-Lyn-p85 complex, which promoted CRC metastasis by increasing PI3K/Akt/β-catenin signaling in tyrosine kinase Lyn-dependent manner. Furthermore, activation of DC-SIGN promoted the transcription of MMP-9 and VEGF by increasing PI3K/Akt/β-catenin signaling, and induced TCF1/LEF1-mediated suppression of miR-185. Our findings reveal the presence of the DC-SIGN–TCF1/LEF1–miR-185 loop in cancer cells with metastatic traits, implying that it may represent a new pathogenic mechanism of CRC metastasis. This character of the loop promises to provide new targets for blocking CRC invasive and metastatic activity.

## Introduction

Metastasis, one of the ten cancer hallmarks, is the major cause of colorectal cancer (CRC)-associated mortality [1]. Over 50% of patients with CRC develop metastases during the course of the disease [2]. The metastatic cascade comprises a series of steps to accomplish invasion, migration, dissemination, and colonization of target organs [3]. However, our understanding of the exact molecular mechanisms underlying CRC metastasis remains incomplete.

Growing evidence suggests the potential correlation between C-type lectin receptors and carcinogenesis [4–6]. DC-SIGN, also known as C-type lectins domain family 4 member L, is highly expressed on immature dendritic cells (DCs) [7, 8], and found at medium levels on macrophages and epithelial cells [9–11]. The majority of previous studies about DC-SIGN focused on its physiologic and pathophysiologic roles in immune cells [8, 12, 13]. Immature DC-SIGN^+^ DCs located intratumorally within CRC lead to immune escape of tumor cells by suppressing immature DCs maturation [14]. Although immature DCs play a pivotal role in escaping immunosurveillance, they account for only a very small fraction of all cells in CRC tissue [15, 16]. However, our previous results showed that DC-SIGN levels in tissues dramatically increased in the early stage of CRC patients. In addition, the staining of DC-SIGN in CRC tissues appears flaky rather than spotty, suggesting that the stained cells were probably not all immune cells [17]. We thus assumed that CRC cells may also express DC-SIGN. We have also previously reported that DC-SIGN family members, LSECtin and DC-SIGNR, promoted gastric and colon cancer liver metastasis [18–20]. Indeed, recent studies have verified that DC-SIGNR and LSECtin are expressed in cancer cells, contributing to tumor development and progression [18, 21]. DC-SIGN is located in glycolipid-enriched membrane domains, which are the specific areas in the plasma membrane signaling platforms, and is associated with Lyn in myeloid cells [22]. Lyn is an important member of the Src family kinases and originally characterized in hematopoietic tissues as an essential kinase required for signaling. Recent studies showed that aberrant activation of Lyn is involved in CRC [23]. Thus, it is not of a surprise that DC-SIGN may also be involved in tumor progression. However, whether CRC cells express DC-SIGN and how DC-SIGN participates in CRC progression remain largely unknown.

miRNAs are important regulators in tumor progression and metastasis [24]. According to the datasets, we noticed that miR-185 is frequently downregulated in CRC and appears to be a promising biomarker [25]. miR-185 is involved in the suppression of the malignant process, especially tumor metastasis [26, 27]. Several studies have documented that miRNAs frequently form feedback loops since they themselves are regulated by transcription factors that may, in turn, target directly or indirectly [28, 29]. Recently, some miRNAs have been shown to be directly regulated by the β-catenin/TCF/LEF1 transcription factor complex [30, 31], suggesting miRNAs as a new class of Wnt effector that significantly contributes to the regulatory role of Wnt/β-catenin signaling.

Here we present evidence that the DC-SIGN–LEF1/TCF1–miR-185 feedback loop in CRC cells contributes to cancer progression. This loop is established by β-catenin/TCF1/LEF1-mediated repression of miR-185 and upregulation of DC-SIGN, which promotes and maintains invasiveness and metastasis in CRC.

## Results

### DC-SIGN is frequently upregulated and its positive expression is associated with poor prognosis in CRC

**Fig. 1:**
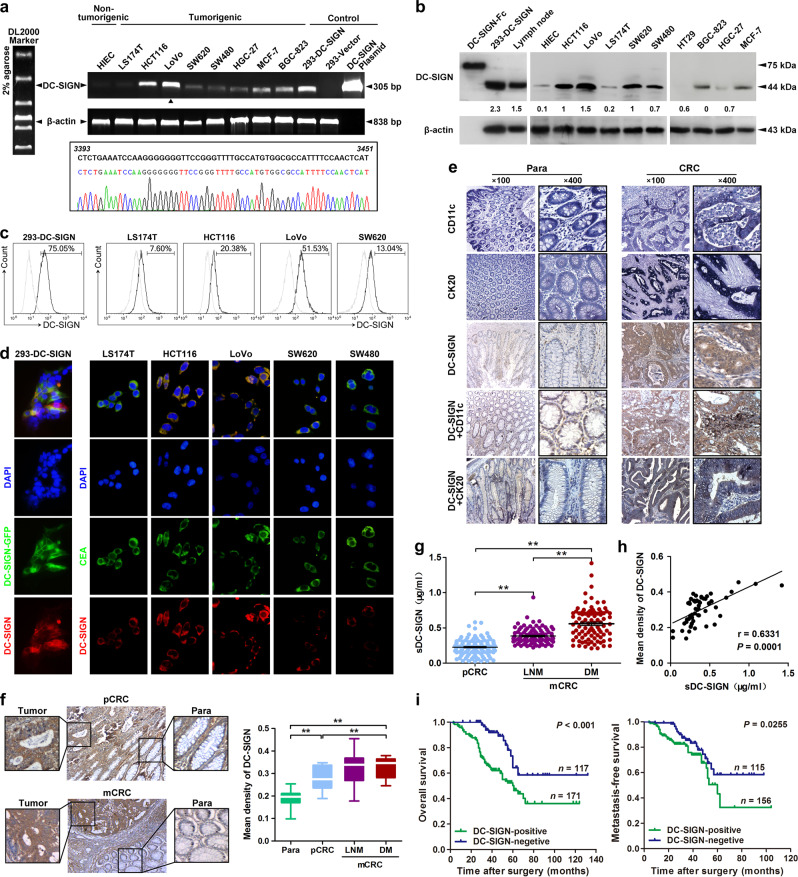
DC-SIGN is frequently upregulated and its positive expression is associated with poor prognosis in CRC. **a** Transcripts expression of DC-SIGN in various human cancer and normal cell lines was detected by standard PCR (upper panel). The PCR products from LoVo cells were confirmed by sequencing (lower panel). **b** Expression of DC-SIGN protein in human CRC. HEK293 transfected with DC-SIGN vector, rhDC-SIGN-Fc, and lymph node lysates were used as positive controls. **c** Cell surface expression of DC-SIGN in colon cancer cell lines was examined by flow cytometry. Numbers listed were percentage of positive cells. Thick line, DC-SIGN; Thin line, isotype control. **d** Confocal microscopy to determine colocalization of DC-SIGN and CEA in colon cancer cells. **e** IHC staining of DC-SIGN and CD11c or cytokine 20 coexpression in CRC tissue. **f** Images shown are representative of DC-SIGN staining in primary (pCRC) and metastatic CRC (mCRC). Para, paracarcinoma; LNM, lymph node metastasis; DM, distant metastasis. **g** Soluble DC-SIGN (sDC-SIGN) levels in serum derived from CRC patients. **h** The correlation between the tissue and serum DC-SIGN expression in matched CRC patients. **i** Kaplan–Meier analysis of the overall survival and metastasis-free survival of CRC patients. Data, mean ± SD. **P *<* 0.01

To investigate the roles of DC-SIGN in CRC, we first identified DC-SIGN expression in different cell lines. By PCR, western blot, and flow cytometry analyses, we found that DC-SIGN displayed different expression levels in a variety of epithelial cancer cells, and its expression levels in CRC cells LoVo and HCT116 are higher than any other cell lines (Fig. 1a–c). DNA sequencing of the PCR products from LoVo cells further validated the DC-SIGN expression, and was identical with the DC-SIGN sequence in GenBank *NM_021155* (Fig. 1a). Confocal assay not only indicated that DC-SIGN costained with CEA, a marker of CRC cells, but also confirmed the surface expression of DC-SIGN in CRC cells (Fig. 1d). Importantly, strong DC-SIGN expression was detected in human CRC tissues. The fact that CRC tissues often contain infiltrated DCs that expressed DC-SIGN implies a possibility that the DC-SIGN-expressing cells are DCs in the CRC tissues. However, immunohistochemistry (IHC) double staining demonstrated that a large portion of DC-SIGN-positive cells costained with cytokine 20 or CD11c, which are the two markers of CRC cell and DCs, respectively, indicating that DC-SIGN does not only express on infiltrated DCs of mesenchyme but also on CRC cells (Fig. 1e).

More importantly, DC-SIGN expression in CRC tissues with metastasis were higher than those without metastasis (Fig. 1f). In addition to the form found on the membrane surface, a soluble form of DC-SIGN (sDC-SIGN) has been previously reported [32]. By ELISA analysis, we detected a significantly higher concentration of sDC-SIGN in CRC patients with distant metastasis than those only with nonmetastatic patients (Fig. 1g). ROC curves indicated that DC-SIGN had good diagnostic accuracy for metastatic CRC (mCRC), and the optimal cutoff was 0.3004 μg/ml (Supplementary Fig. S1a). Moreover, sDC-SIGN had greater diagnostic efficacy than CEA in patients with mCRC (Supplementary Fig. S1b, c). Of note, tissue expression of DC-SIGN was positively correlated with sDC-SIGN expression (Fig. 1h). DC-SIGN-positive expression in CRC tissues was significantly associated with a more aggressive tumor phenotype (Supplementary Table S1). Furthermore, the patients with positive DC-SIGN had shorter overall and metastasis-free survival (Fig. 1i). DC-SIGN expression, as well as metastasis and TNM stage, were independent prognostic factors for the survival of CRC patients (Supplementary Table S2). These results suggest that DC-SIGN is expressed in CRC cells, and its expression levels predict poor prognosis in mCRC patients.

We also analyzed the genomic alterations of DC-SIGN gene in 276 CRC cases from The Cancer Genome Atlas project reported previously [33]. In total, 4 of 276 cases were found to carry missense mutations (G55E, E93D, and A283T) and a truncating mutation (X36_splice), and another two cases had DNA copy number amplification (Supplementary Fig. S2a and Supplementary Table S3). The three missense mutations showed little effect on DC-SIGN protein levels or DC-SIGN/Lyn interactions (Supplementary Fig. S2b).

### DC-SIGN silencing inhibits CRC-cell growth and metastasis in vitro and in vivo

**Fig. 2:**
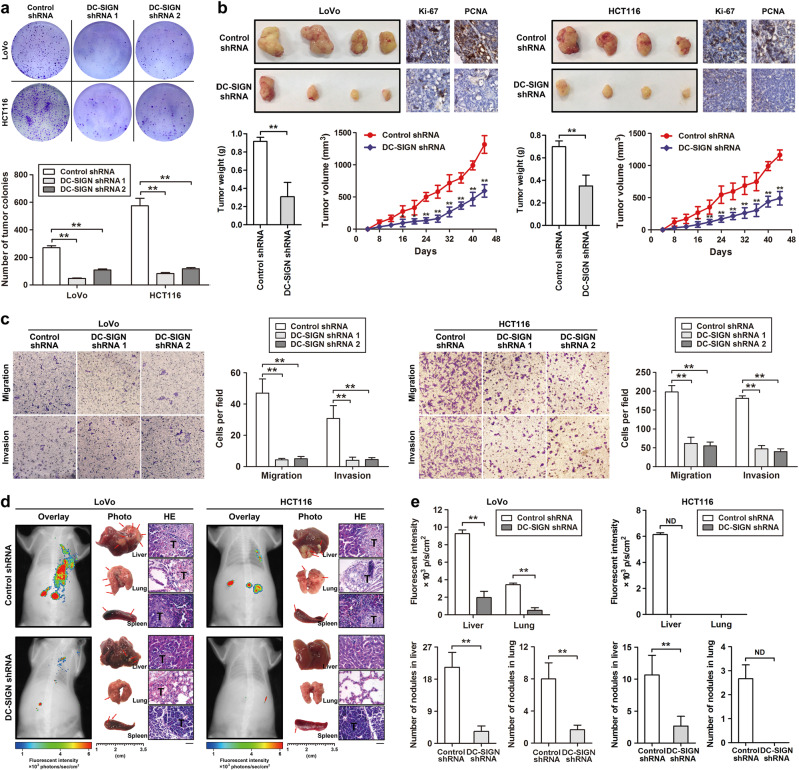
DC-SIGN silencing inhibits the growth and metastasis of CRC cells in vitro and in vivo. **a** Stable CRC cells expressing control shRNA or DC-SIGN shRNA were applied to colony formation analysis. **b** Growth of subcutaneous xenografts from DC-SIGN shRNA and control cells. **c** Representative images of LoVo and HCT116 migration and invasion of cells on the membrane. **d** Fluorescence analysis of the kinetics of metastases of the indicated cells (left panel). The spleens, livers, and lungs were examined at the gross anatomical level for tumors and metastases (middle panel). Representative H&E staining showing metastatic nodules (right panel). T, tumor. **e** Fluorescent intensity and quantitation of micrometastases per liver and lung as assessed. Data, mean ± SD. *P < 0.05, **P < 0.01. N.D., not determined

To investigate whether DC-SIGN is involved in CRC progression, two DC-SIGN shRNAs (DC-SIGN shRNAs 1 and 2) were designed to silence DC-SIGN expression via lentivector transduction (Supplementary Fig. S3a, b), both of which efficiently inhibited DC-SIGN expression in LoVo and HCT116 cells (Supplementary Fig. S3c). Methyl-thiazolyltetrazolium (MTT) and colony formation assays revealed the proliferation-repressing function of DC-SIGN knockdown in CRC cells (Fig. 2a and Supplementary Fig. S3d). Furthermore, in vivo analysis showed that silencing of DC-SIGN in CRC cells caused dramatic reductions in tumor weight and volume in nude mice. Such effect was further confirmed by measuring the expression of Ki-67 and PCNA (Fig. 2b). Transwell and wound-healing assays demonstrated that silencing of DC-SIGN markedly decreased the migratory and invasive potential of tumor cells (Fig. 2c and Supplementary Fig. S3e). In addition, we injected stable CRC cells into the distal tip of the spleen and examined tumor metastasis in vivo by monitoring GFP fluorescent intensity, and found that fluorescence tumor signal was decreased in mice bearing DC-SIGN-depleted tumor cells (Fig. 2d, e, upper panel). After the killing of mice, we noticed that metastatic lesions detected microscopically were fewer and smaller in the livers and lungs of nude mice inoculated with CRC cells transfected with DC-SIGN shRNA compared with control (Fig. 2e, lower panel). IHC staining results further confirmed that the metastatic lesions had replaced large areas of liver and lung parenchyma (Supplementary Fig. S4). Together, these results suggest that DC-SIGN can significantly promote cell growth and metastasis in vitro and in vivo.

### miR-185 suppresses MMP-9 and VEGF expression and metastatic potential of CRC cells by targeting DC-SIGN

**Fig. 3:**
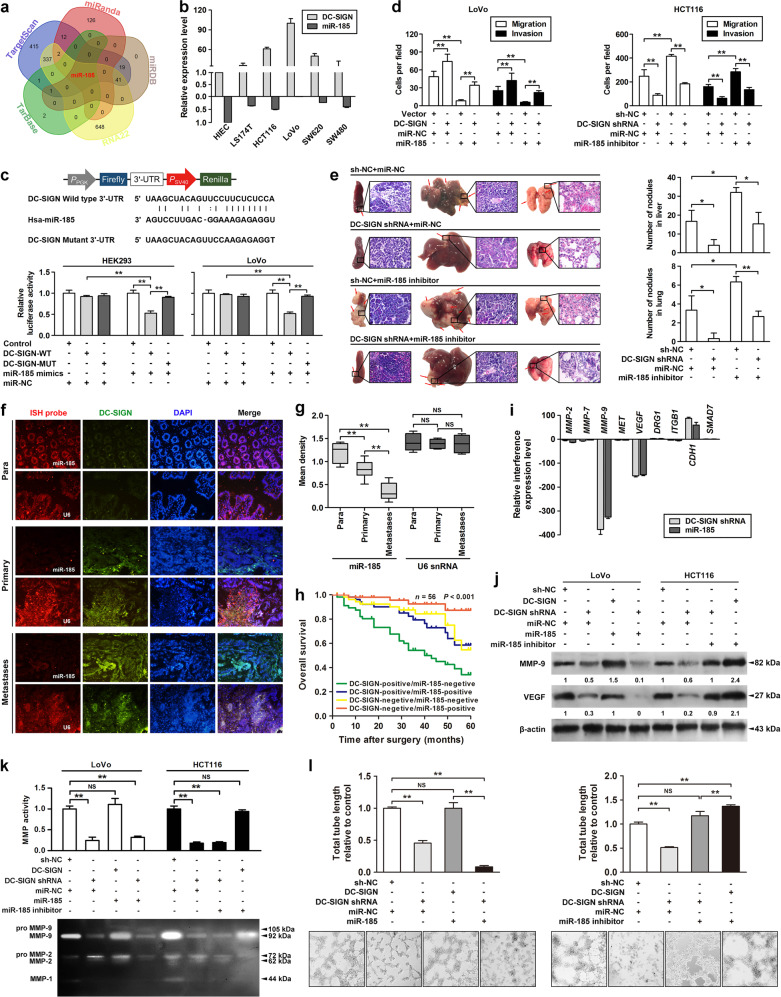
miR-185 suppresses MMP-9 and VEGF expression and metastatic potential of CRC cells by targeting DC-SIGN. **a** Five independent miRNA target databases were used to predict the potential miRNAs. **b** Endogenous expressions of DC-SIGN and miR-185 in various human CRC cells were detected by qPCR. **c** Luciferase activity assay for targeting sequences of the 3′-UTR of DC-SIGN by miR-185 in HEK293 and LoVo cells. **d** Cells were transfected as indicated, and applied to transwell analysis. **e** The spleens, livers, and lungs were examined for tumors and metastases of the indicated cells (left panel). Quantitation of micrometastases per liver and lung as assessed (right panel). **f** Representative images of fluorescent in situ hybridization and immunofluorescence of miR-185 and DC-SIGN in paired CRC tissues. Para, paracarcinoma. U6 snRNA was used as a control. **g** Expression of miR-185 and U6 were semiquantitative by ISH analysis. **h** Kaplan–Meier curves for all patients divided by combination of miR-185 and DC-SIGN status. **i** Relative mRNA levels of CRC metastasis-related genes in DC-SIGN-depleted LoVo cells. **j** The lysates of stable LoVo and HCT116 cells were applied to western blot. **k** Culture supernatants of CRC cells were applied to gelatin zymography. **l** Representative images of stable LoVo and HCT116 tube formation of cells on the Matrigel. Data, mean ± SD. *P *<* 0.05, **P *<* 0.01. N.S., nonsignificant

miRNAs are important regulators of cancer. To investigate whether miRNA participates in the regulation of DC-SIGN expression, we used five independent databases to predict miRNAs that may be involved (Fig. 3a). miR-185 was identified as an ideal miRNA with the highest prediction score and was highly conserved in mammals (Supplementary Table S4 and Fig. 5a). The miR-185 binding sites were located in ‘unstable’ regions with multibranching loops of DC-SIGN mRNA (Supplementary Fig. S5b) [34]. Therefore, we examined the levels of miR-185 and DC-SIGN in several CRC-cell lines by qPCR (Fig. 3b), and found that the endogenous DC-SIGN and miR-185 levels were inversely correlated (Supplementary Fig. S5c). Moreover, cotransfection of miR-185 markedly decreased DC-SIGN-WT 3′-UTR luciferase activity (Fig. 3c). miR-185-induced downregulation of DC-SIGN in LoVo cells was rescued by re-expression of DC-SIGN, and that upregulation of DC-SIGN in HCT116 cells by miR-185 inhibitor was partially reversed by silencing DC-SIGN (Supplementary Fig. S5d). Transwell, wound-healing, MTT, and colony formation assays all indicated that miR-185 inhibitor could functionally restore metastatic capacity and proliferation activity of DC-SIGN silence-suppressed HCT116 cells (Fig. 3d and Supplementary Fig. S6). Furthermore, in vivo analysis demonstrated that the number of metastatic nodules in the liver and lung were decreased after being inoculated with the DC-SIGN-silenced HCT116 cells, and were largely restored by miR-185 inhibitor (Fig. 3e). In addition, we performed fluorescence in situ hybridization for analyzing the expression of DC-SIGN and miR-185 in CRC tissues. Compared to normal tissues, the miR-185 levels were significantly reduced in matched CRC tissues (Fig. 3f). The low miR-185 expression levels were associated with CRC metastasis (Fig. 3g). Notably, the patients with both low miR-185 expression and high DC-SIGN expression had a shorter overall 5-year survival time than all other combined status patients (Fig. 3h).

To facilitate a metastasis, tumor cells must complete a multistep progression through producing cell-adhesion molecules, matrix degradation enzymes, and vascular growth factors [35]. Among these metastasis-associated molecules, the mRNA and protein levels of MMP-9 and VEGF were significantly decreased in DC-SIGN-depleted LoVo cells (Fig. 3i, j). Functional studies also exhibited that miR-185 downregulation abrogated the changes in the gelatin zymography and tube formation of tumor cells induced by DC-SIGN knockdown (Fig. 3k, l). In addition, miR-185 restoration could reverse DC-SIGN protein levels and the downstream molecular changes induced by DC-SIGN-WT (Supplementary Fig. S7a). Ectopic DC-SIGN expression totally rescued the invasion and migration of LoVo cells from miR-185-mediated suppression (Supplementary Fig. S7b). Together, these results suggest that miR-185 is a critical upstream mediator of DC-SIGN promoting CRC metastasis.

### miR-185/DC-SIGN signaling blocks β-catenin translocation through the PI3K/Akt/GSK-3β pathway

**Fig. 4:**
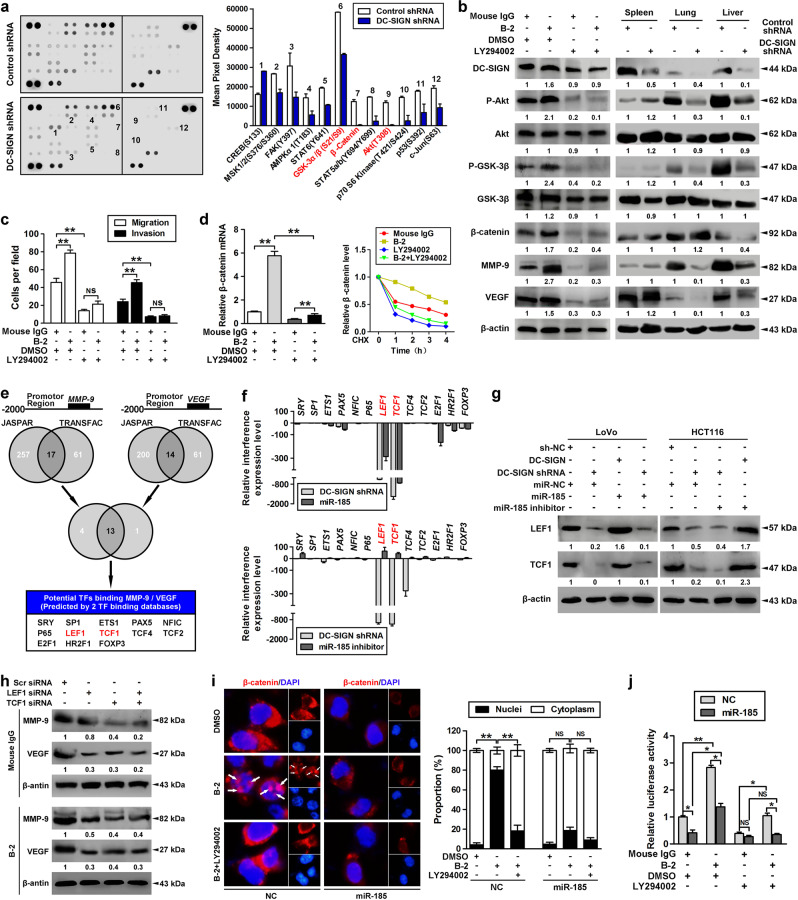
miR-185/DC-SIGN signaling blocks β-catenin translocation through the PI3K/Akt/GSK-3β pathway. **a** The lysates of stable LoVo cells infected with Lenti-DC-SIGN shRNA or control were applied to phosphokinase antibody array, and pixel densities of indicated proteins were shown. **b** LoVo cells were treated with DC-SIGN agonistic antibody (B-2, 10 μg/ml) for 15min and/or PI3K inhibitor (LY294002, 50 μM) for 24h, followed by western blot analysis (left panel). Stable LoVo cells expressing control shRNA or DC-SIGN shRNA were injected into the spleen. The lysates of spleen, lung, and liver tissues were applied to western blot (right panel). **c** LoVo cells were treated with DC-SIGN mAb and/or LY294002, and applied in transwell analysis. **d** Total RNA was extracted and applied to qPCR (left panel), or cells were further pulsed with CHX (20 μM) and applied to western blot (right panel). **e** Two independent TFs target databases were used to predict the potential TFs binding to promotors of MMP-9 and VEGF. **f** Interference levels of indicated TFs were shown by qPCR. **g** Cells were transfected as indicated, and lysates were applied to western blot. **h** LoVo cells expressing control LEF1 and/or TCF1 siRNA were treated with nonspecific IgG or DC-SIGN mAb. Then cell lysates were applied in western blot analysis. **i** Immunofluorescent analysis of β-catenin subcellular distribution in LoVo cells transfected with miR-185 or control while being treated with DC-SIGN mAb and/or LY294002 (left panel). Quantitation of β-catenin distribution (right panel). **j** LoVo cells were treated as indicated, and applied to luciferase-based β-catenin/TCF transcriptional activity assay (TOPflash/FOPflash). Data, mean ± SD. *P *<* 0.05, **P *<* 0.01

We next evaluated how miR-185/DC-SIGN signaling modulated CRC metastasis. By using a human phosphokinase antibody array, we screened twelve differentially regulated signaling molecules in DC-SIGN-depleted LoVo cells, among which phospho-Akt (T308), phospho-GSK-3α/β (S21/S9), and β-catenin levels were significantly decreased (Fig. 4a). Because previous research indicated that DC-SIGN-mediated signaling involved tyrosine phosphorylation [12], we determined whether DC-SIGN activation in the CRC cell led to tyrosine-dependent signaling. The antibody B-2, which recognizes an epitope in the stalk of the DC-SIGN extracellular domain, can effectively activate DC-SIGN (Supplementary Fig. S8a). Stimulation with DC-SIGN agonistic antibody increased levels of phospho-Akt/GSK-3β and β-catenin in LoVo cells, and this effect was reversed by PI3K inhibitor LY294002 (Fig. 4b, left panel). In addition, in vivo analysis revealed that silencing of DC-SIGN could inactivate PI3K/Akt/GSK-3β/β-catenin signaling and reduce MMP-9 and VEGF expression (Fig. 4b, right panel). Transwell assay demonstrated that administration of LY294002 dramatically repressed DC-SIGN mAb-induced tumor migration and invasion in vitro (Fig. 4c). Because previous studies indicated that Akt and ERK can phosphorylate GSK-3β, resulting in decreased stability and expression of β-catenin [36–38]. By blocking protein synthesis with cycloheximide, we found that LY294002 abolished the increased mRNA level and protein stability of β-catenin in DC-SIGN-agonistic LoVo cells (Fig. 4d). However, administration of ERK inhibitor U1026 failed to block DC-SIGN mAb-mediated β-catenin, MMP-9, and VEGF upregulation (Supplementary Fig. S8b). These results suggest that PI3K/Akt signaling activation is critical for DC-SIGN-induced cell metastasis in CRC.

Although β-catenin contains no DNA-binding domain, it can directly impact gene expression if it translocates to the nucleus and is recruited to chromatin via interaction with DNA-binding TCF/LEF family proteins [39]. Analysis of the transcription factor-binding profiles identified that, within −2000–0 bp of MMP-9 and VEGF promotors contain specific binding sites of TCF/LEF1 (Fig. 4e). Among a series of transcription factors, DC-SIGN knockdown significantly suppressed TCF1 and LEF1 expression (Fig. 4f, g). We next transfected LoVo cells with TCF1 and/or LEF1 siRNA, and found that stimulation with DC-SIGN mAb cannot promote MMP-9 and VEGF expression (Fig. 4h). Interestingly, β-catenin is redistributed to the nucleus in ~78% of the cell population following DC-SIGN mAb treatment, whereas upon treatment with LY294002 or transfection with miR-185, the nuclear distribution of β-catenin significantly decreased (Fig. 4i). Furthermore, LY294002 suppressed the β-catenin/TCF/LEF-dependent promoter activity during DC-SIGN mAb stimulation (Fig. 4j). These results suggest that miR-185/DC-SIGN signaling suppresses β-catenin translocation of CRC cells through inactivating the PI3K/Akt/GSK-3β pathway.

### DC-SIGN promotes CRC metastasis through PI3K/Akt/β-catenin activation in tyrosine kinase Lyn–dependent manner

**Fig. 5:**
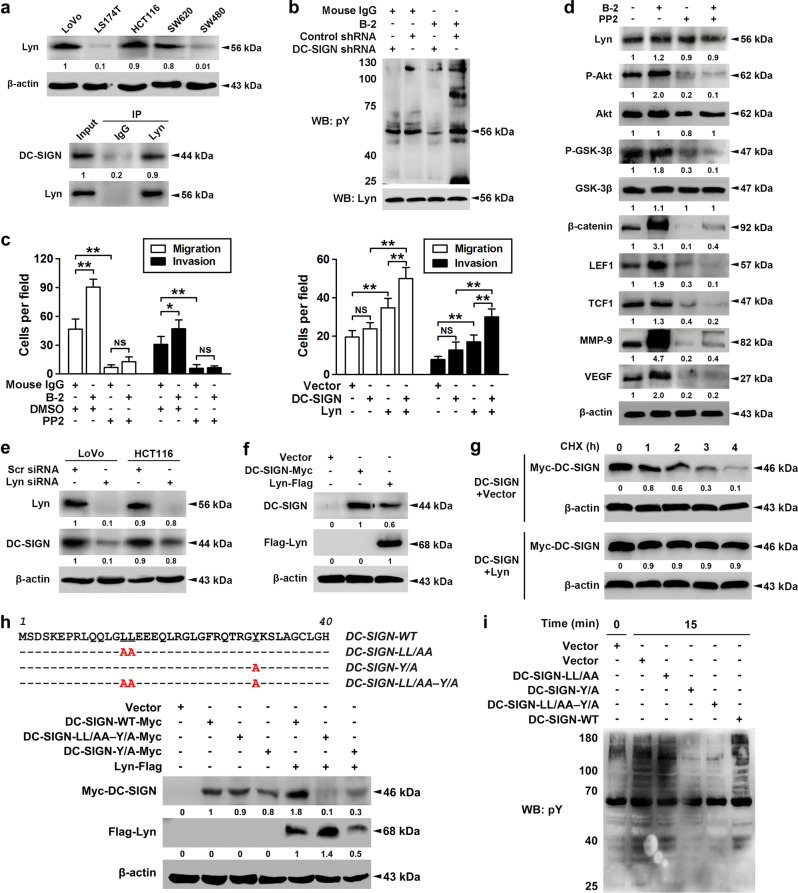
DC-SIGN promotes the CRC metastasis through PI3K/Akt/β-catenin activation in tyrosine kinase Lyn-dependent manner. **a** The expression profiles of Lyn in CRC cells were shown in upper panel, and immunoprecipitation using Lyn antibody was performed in LoVo cell lysates (lower panel). **b** Stable LoVo cells treated with nonspecific IgG or DC-SIGN mAb (B-2, 10 μg/ml) were collected, lysed, and cell lysates were applied to immunoprecipitation with Lyn antibody. **c** LoVo cells treated with DC-SIGN mAb and/or PP2 (50 μM, left panel) and LS174T cells infected with DC-SIGN and/or Lyn vectors (right panel), and applied in transwell analysis. **d** LoVo cells were treated with DC-SIGN mAb and/or PP2. Then cell lysates were applied in western blot analysis. **e** LoVo and HCT116 cells were transiently transfected scramble or Lyn siRNA, and lysates were applied to western blot. **f** LS174T cells were transfected with vector, Lyn, or DC-SIGN construct, and lysates were applied to western blot. **g** LS174T cells were transfected as indicated, pulsed with cycloheximide (CHX, 20 μM), and applied to western blot. **h** Amino acid sequence alignment of the cytoplasmic domain of WT and mutant DC-SIGN proteins (upper panel). Red letters identify amino acid substitutions. LS174T cells were transfected with constructs as indicated, followed by western blot (lower panel). **i** After transfection, cells were treated with DC-SIGN mAb, and lysates were applied to western blot. Data, mean ± SD. *P *<* 0.05, **P *<* 0.01. N.S., nonsignificant

Previous research indicated that DC-SIGN in DCs coprecipitates with the tyrosine kinases Lyn [22]. Similarly, Lyn was found to interact with endogenous DC-SIGN in LoVo cells (Fig. 5a). The mRNA and protein levels of Lyn were much higher in LoVo and HCT116 cells (Fig. 5a and Supplementary Fig. S9a). In addition, stimulation with DC-SIGN mAb increased Lyn tyrosine phosphorylation, whereas DC-SIGN knockdown abrogated Lyn activation (Fig. 5b). Functional studies also exhibited that activation of DC-SIGN signaling promoted the migration and invasion in LoVo cells, and the promotive effect was abolished by Lyn inhibitor PP2 (Fig. 5c, left panel). Inhibition of Lyn blocked DC-SIGN mAb-mediated phospho-Akt/GSK-3β upregulation as well as the increase in MMP-9 and VEGF expression (Fig. 5d). Cotransfection of DC-SIGN and Lyn in LS174T cells induced a more significant promotive effect on tumor migration and invasion compared with transfection with Lyn or DC-SIGN alone (Fig. 5c, right panel).

We then evaluated whether and how DC-SIGN and Lyn were coordinately expressed in CRC. Lyn knockdown or overexpression could significantly affect the protein expression of DC-SIGN (Fig. 5e, f), but had little effect on the mRNA levels (Supplementary Fig. S9b). Indeed, cotransfection with Lyn promoted the protein stability of exogenous DC-SIGN in LS174T cells (Fig. 5g). Furthermore, transfection of wild- or mutant-type DC-SIGN alone exhibited faint expression, but cotransfection with Lyn remarkably promoted the expression of wild-type DC-SIGN, though not much effectively enhanced the protein levels of Y/A and LL/AA–Y/A mutant (Fig. 5h). Because previous reports indicated that DC-SIGN signaling requires Src family kinases, and tyrosine residue and dileucine motif in the DC-SIGN cytoplasmic tail are associated with transmission of intracellular signals [12, 22], we assumed that tyrosine residue and dileucine motif may be required for Lyn to stimulate DC-SIGN expression. We stimulated each of the transfected cells with DC-SIGN mAb, and found that loss of signaling occurred exclusively in LL/AA–Y/A mutant (Fig. 5i), which suggested a critical function of the tyrosine residue and dileucine motif in the DC-SIGN cytoplasmic tail for the transmission of intracellular signals.

Lyn is reported to be able to regulate PI3K activity and Akt activation through interaction with p85 [40]. We examined whether PI3K was physically associated with Lyn in the presence of DC-SIGN mAb stimulation, and found that DC-SIGN coprecipitated preferentially with p85 and Lyn. Likewise, DC-SIGN and p85 protein levels in the immunoprecipitates of Lyn were also dramatically upregulated following DC-SIGN mAb treatment (Supplementary Fig. S9c). In addition, we examined whether Lyn is associated with Akt, GSK3β, metastases-related TFs, and target proteins to modulate their expression or activation. However, no interactions were found between Lyn and these proteins (Supplementary Fig. S9d). These results suggest that Lyn is certainly involved in mediating DC-SIGN activation to PI3K/Akt/β-catenin signaling.

### β-catenin/TCF1/LEF1 directly suppresses miR-185 expression in CRC cells

**Fig. 6:**
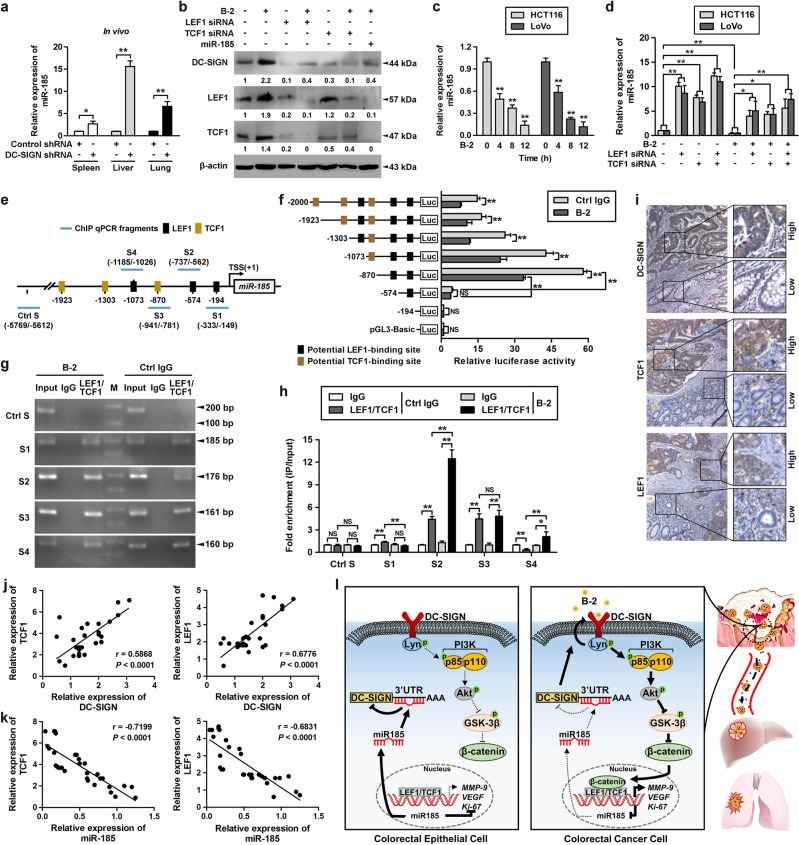
β-catenin/TCF1/LEF1 directly suppresses miR-185 expression in CRC cells. **a** Stable LoVo cells expressing control shRNA or DC-SIGN shRNA were injected into the spleen. Then total RNA of spleen, lung, and liver tissues was extracted and applied to qPCR. **b** Cells were transfected as indicated and treated with DC-SIGN mAb (B-2, 10 μg/ml), followed by western blot. **c** Cells were treated with DC-SIGN mAb, and expression of miR-185 was examined using qPCR. **d** Cells infected with LEF1 and/or TCF1 siRNA were treated with DC-SIGN mAb, and total RNA was extracted and applied to qPCR. **e** Schematic presentation of the genomic localization of the human miR-185 gene and its promoter region. **f** Serially truncated miR-185 promoter constructs were cloned to pGL3-luciferase reporter plasmids and transfected into LoVo cells. Cells were treated with DC-SIGN mAb, and applied in luciferase activities analysis. **g** Chromatin of LoVo cells cultured in the absence and presence of DC-SIGN mAb was subjected to chromatin immunoprecipitation with antibodies against LEF1 or TCF1 followed by PCR analysis using primers amplifying different regions of the miR-185 promoter indicated in **d**. M, DL1000 Marker. **h** qPCR of the ChIP products validated the binding capacity of LEF1 and TCF1 to the miR-185 promoter. **i** Representative images of IHC staining for DC-SIGN, LEF1, and TCF1 in CRC tissue. **j, k** Correlation between LEF1 or TCF1 expression and DC-SIGN (**j**) or miR-185 (**k**) levels in CRC samples. Spearman correlation coefficient with the respective significance is indicated. **l** Schematic representation of the proposed roles of DC-SIGN–LEF1/TCF1–miR-185 feedback loop in the of CRC progression. Data, mean ± SD. *P *<* 0.05, **P *<* 0.01. N.S., nonsignificant

Given that miR-185 is significantly downregulated in CRC, we were interested to identify the molecular mechanisms that regulate miR-185 expression. By RT-PCR analysis, we found that the expression of miR-185 was markedly increased in the metastasis model of DC-SIGN silencing (Fig. 6a). Interestingly, DC-SIGN expression was induced by DC-SIGN agonistic antibody and this expression could be blocked by knockdown of TCF1/LEF1 and restoration of miR-185 (Fig. 6b), indicating that TCF1/LEF1 regulates DC-SIGN through miR-185. miR-185 expression decreased in a time-dependent manner in LoVo cells treated with DC-SIGN mAb (Fig. 6c). Moreover, knockdown of TCF1/LEF1 prevented the repression of miR-185 after DC-SIGN mAb treatment (Fig. 6d). Thus, we evaluated whether TCF1/LEF1 could in turn target miR-185 directly during CRC progression. The promoter region of miR-185 was predicted to be chr22: 20018662-20020743 in the UCSC database, and six potential TCF1/LEF1-binding sites were identified by JASPAR (Fig. 6e, Supplementary Table S5). Truncation mutations of these binding sites revealed that the locus of −589 to −575 bp upstream of the transcriptional start site is the major site for TCF1/LEF1 repression of miR-185 transcriptional activity (Fig. 6f). We next performed chromatin immunoprecipitation for endogenous TCF1/LEF1 in DC-SIGN mAb agonistic LoVo cells, followed by qPCR analysis with various primer pairs covering different regions of the miR-185 promoter (Fig. 6g, h). This experiment further confirmed that TCF1/LEF1 binds to the same site of the promoter of miR-185. Notably, TCF1/LEF1 binding were only observed in cancer cells and not in their epithelial counterparts (Supplementary Fig. S10). Together, these results suggest that the DC-SIGN/TCF1/LEF1 pathway suppresses miR-185 transcription in CRC cells.

### The DC-SIGN–LEF1/TCF1–miR-185 feedback loop is the characteristic of human CRC samples

To determine the clinical relevance of our observations, we examined the expression of the DC-SIGN, LEF1, and TCF1 during progression of CRC (Fig. 6i). We found that elevated DC-SIGN levels tended to increase the expression of LEF1 and TCF1 in CRC sections (Fig. 6j). Combined with the previous data, we found that miR-185 levels and TCF1 or LEF1 levels exhibited a strong negative correlation (Fig. 6k), indicating that the reduced miR-185 expression resulted at least partially from elevated TCF1 and LEF1 expression. In summary, these results strongly suggest that the DC-SIGN–LEF1/TCF1–miR-185 feedback loop is highly active in human CRC and promotes invasion and metastasis.

## Discussion

DC-SIGN was first cloned from a human placental cDNA library and later found to be expressed on the surface of both DCs and macrophages [8, 9]. In this study, we identified expression of DC-SIGN in CRC cells by using several techniques. We surprisingly found that DC-SIGN was highly expressed in CRC cells and frequently upregulated in patients with CRC metastases. In addition, loss of DC-SIGN inhibited malignant capacities of CRC cells by suppressing invasion and migration, proliferation, and angiogenesis. These data imply that DC-SIGN may play a critical role in promoting CRC metastasis. It is consistent with previous findings that related members of CD209 family such as DC-SIGNR, LSECtin, and CD23 are expressed in cancer cells, contributing to tumor progression [18, 21, 41].

The upregulation of DC-SIGN in CRC cells has rarely been understood. In this study, the mechanism of DC-SIGN upregulation in CRC was mainly elucidated in the following two aspects. First, we found miR-185 targets DC-SIGN mRNA by binding to the DC-SIGN 3′-UTR to downregulate DC-SIGN expression, which confirmed previous findings that miR-185 regulates gene expression by initiating mRNA degradation and functions as a tumor suppressor [26, 27]. We also found that the low miR-185 expression level was associated with CRC metastasis. Furthermore, miR-185 level was inversely correlated with DC-SIGN level in both CRC cells and tissues. This might be a possible explanation for the overexpression of DC-SIGN in CRC cells. Second, our findings revealed Lyn could promote DC-SIGN expression at posttranscriptional level. Considering that Lyn is a classical receptor dependent protein [22, 42], we hypothesizes that Lyn could interact with endogenous DC-SIGN. This was supported by our results that Lyn and DC-SIGN achieve coexpression in CRC cells through forming a complex. In addition, overexpression of Lyn in turn increased the protein level and stability of DC-SIGN. Hence, these results at least partly explained why DC-SIGN is upregulated in CRC.

The molecular mechanism of DC-SIGN in CRC metastasis is poorly understood to date. In this study, we found that DC-SIGN/Lyn signaling increased MMP-9 and VEGF expression in CRC by promoting PI3K/Akt activation and nuclear translocation of β-catenin. Moreover, miR-185 expression was reduced following DC-SIGN agonistic antibody treatment and that silencing TCF1/LEF1 could abrogate this reduction. This was of particular interest to us given that β-catenin/TCF1/LEF1 signaling, in addition to being implicated in target gene, has been shown recently to play a key role in the transcription of certain miRNA [30, 31]. Here, we demonstrated that β-catenin/TCF1/LEF1 activation in CRC suppresses miR-185 transcription. We also found DC-SIGN expression positively correlated with TCF1/LEF1 expression but negatively correlated with miR-185 expression in human CRC samples, which verified the feedback loop between DC-SIGN, TCF1/LEF1, and miR-185 interregulation. Notably, several known targets of the components of the DC-SIGN–TCF1/LEF1–miR-185 loop might be important for CRC progression. Recent studies showed that inhibition of GSK-3β activity induces DC-SIGN expression [43], thereby providing additional feedback that may reinforce the loop. Others found that the transcription of Wnt target gene c-myc [44], an miR-185 functional target [45], was increased by the activation of DC-SIGN [46], suggesting another potential feedback loop that may regulate CRC. Thus, further work is warranted to elucidate this complex network.

## Materials and methods

### Clinical samples

With approval and support from the ethics committee of the Second Affiliated Hospital of Dalian Medical University. CRC tissues and serum were obtained from the Second Affiliated Hospital of Dalian Medical University. The diagnosis of colorectal carcinoma was confirmed by pathologic examination. Informed consent was obtained from all patients.

### Cell culture and reagents

Human CRC (LS174T, HCT116, LoVo, SW620, SW480, and HT29), gastric cancer (HGC-27 and BGC-823), breast cancer (MCF-7), and HEK293 cell lines were purchased from the Institute of Biochemistry and Cell Biology of the Chinese Academy of Sciences (Shanghai, China), and normal human intestinal epithelial cells were obtained from the Xijing Hospital of Digestive Diseases (Xi'an, China). Cells were cultured in RPMI 1640 Medium or Dulbecco’s Modified Eagle’s Medium supplemented with 10% FBS and 100 U/ml penicillin/streptomycin at 37 °C in a humidified incubator containing 5% CO_2_. Recombinant human DC-SIGN-Fc (Sino Biological) was dissolved in PBS containing 0.1% BSA. Cells were stimulated with DC-SIGN agonistic antibody (Santa Cruz) at a concentration of 10 μg/ml or mouse immunoglobulin G (Proteintech). LY294002, PP2, and U0126 were obtained from Selleck and used at a final concentration of 50 μM. Cycloheximide (Sigma-Aldrich) was dissolved in DMSO (100 mM stock solution) and always used at a final concentration of 20 μM.

### Plasmids construction, siRNA treatment, and lentivirus transduction

DC-SIGN-Myc/His and Lyn-Flag expression plasmids were produced based on the full coding sequence and synthesized by GeneChem (Shanghai, China). The DC-SIGN LL-AA and/or Y-A mutant was generated using the mutanBEST kit (Takara) following the manufacturer’s instructions. The lentiviral plasmids pGLV3 expressing miR-185, miR-185-sponge inhibitor, or the negative control sequences were purchased from GenePharma. The siRNAs specific for Lyn, LEF1 or TCF1, and their control siRNA were purchased from Santa Cruz. Transient transfections were carried out using Lipofectamine 2000 (Invitrogen) following the manufacturer’s protocol. For stably knocking down DC-SIGN, cells were infected with DC-SIGN short hairpin RNA (shRNA) lentiviral plasmids, and clones were selected with 2 mg/ml puromycin (Sigma-Aldrich).

### Double-staining immunohistochemistry

Tissue samples were cut at a thickness of 4 μm, dewaxed in xylene, and gradually rehydrated with gradient alcohol. Antigen retrieval was performed by heating the slides in 0.01 M citrate buffer (pH 6.0) for 30 min. Endogenous phosphatase was blocked with 3% acetic acid, and the slides were then incubated overnight at 4 °C with the primary antibody (CD11c 1:100, Proteintech, CK20 1:100, Bioworld). Following incubation with the primary antibody, the sections were treated for 20 min with 3% hydrogen peroxide. Then, the slides were incubated with an alkaline phosphatase-conjugated goat anti-mouse antibody (1:800, ZSGB-Bio, China) for 1 h at room temperature (RT), and 5-bromo-4-chloro-3-indolyl phosphate and nitro blue tetrazolium were used for color development. The slides were next incubated overnight at 4 °C with a rabbit anti-human DC-SIGN antibody (1:150, Abcam) and a horseradish peroxidase-conjugated goat anti-rabbit antibody (1:800, ZSGB-Bio) for 1 h, followed by incubation with 3,3-diaminobenzidine and H_2_O_2_, at RT. Visualization and photography were performed using a Leica DM4000B microscope (Leica). The tissues were scored by counting the number of cells with positive staining in 10 separate fields at ×400 magnification.

### Flow cytometry

Approximately 1 × 10^6^ CRC cells were detached using trypsin (HyClone), washed with cold PBS, and incubated with APC anti-DC-SIGN monoclonal antibody (5 μl/test, eBioscience; 5 μl/test, BioLegend) or matched APC isotype control antibody for 2 h at 37 °C. Fluorescence was detected in a NovoCyte Flow Cytometer (ACEA Biosciences) and analyzed with a flow cytometry (NovoCyte; ACEA Biosciences) equipped with a NovoExpress software.

### Immunofluorescence and confocal microscopy analyses

For double labeling immunofluorescence analyses, cells were rinsed twice with cold PBS and fixed in 4% paraformaldehyde for 15 min at RT. The nonspecific sites were blocked by incubation with 5% BSA in PBS for 30 min at RT. Then, cells were incubated with a rabbit anti-human CEA antibody (1:100, Bioworld) diluted in PBS for overnight at 4 °C. After four washes in PBS, cells were coincubated with secondary antibodies conjugated to FITC (1:200, ZSGB-Bio) and anti-human DC-SIGN antibody conjugated to APC (eBioscience) for 1 h at RT, washed and mounted in vectashield containing DAPI (Vector Laboratories). Confocal microscopy was performed with a Leica TCS SP5II scanning confocal microscope (Leica).

### Enzyme-linked immunosorbent assay (ELISA)

The soluble form of DC-SIGN was quantified using an ELISA according to the manufacturer’s recommendations. Briefly, 96-well Nunc-Immuno microtiter plates with a MaxiSorp surface (Thermo Scientific) were coated with 100 μl of a monoclonal antibody to DC-SIGN (1:4000, Sigma-Aldrich) and incubated at 4 °C overnight. The reaction was blocked with 1% bovine serum albumin for 1 h at 37 °C. The plates were incubated with anti-human DC-SIGN antibody (1:2000, Abcam) for 2 h at 37 °C, followed by the addition of 100 μl of a 1:2000 dilution of streptavidin-horseradish peroxidase for 1 h. Color development was achieved by adding 100 μl of 3,3,5,5-tetramethyl-benzidine as a substrate to each well. Sulfuric acid (1 mol/L) was added to stop the reaction. The optical density was measured at 450 nm and referenced to 570 nm using a Multiskan FC multimode plate reader (Thermo Scientific).

### Immunoprecipitation and western blot

Immunoprecipitation assays were performed using the Crosslink Magnetic IP/Co-IP Kit (Thermo Scientific). Cells were solubilized with immunoprecipitation buffer, equal amounts of protein were incubated with specific antibody immobilized onto protein A/G magnetic beads overnight at 4 °C with gentle rotation. Beads were washed extensively with immunoprecipitation lysis buffer, resuspended, and eluted. Protein samples were subjected to SDS-PAGE followed by western blot analysis.

For western blot assay, total protein was extracted from the cultured cells using the total protein extraction kit (KeyGene). The protein content was determined using the bicinchoninic acid protein assay kit (Bio-Rad) with bovine serum albumin as the standard. Proteins (30 μg) were separated by 8–12% SDS–PAGE and transferred onto nitrocellulose filter membranes (Life Science). After blocking in 5% nonfat milk in Tris-buffered saline with 0.05% Tween-20, the membranes were probed with primary antibodies overnight at 4 °C followed by washing and incubation with horseradish peroxidase-conjugated secondary antibodies for 2 h at RT. Antigen-antibody complexes were visualized using enhanced chemiluminescence detection kit (Advansta) and image analyzer ImageQuant LAS 500 (GE Healthcare).

### Quantitative real-time PCR

Total RNA was extracted using TRIzol (Invitrogen). The concentrations of RNA were determined using a NanoDrop 2000 (Thermo Scientific). For mRNA and miRNA analyses, cDNA was generated from 2 μg total RNA per sample using the PrimerScript RT Reagent Kit (Takara) and miRcute Plus miRNA First-Strand cDNA Kit (TIANGEN), respectively. qPCR primers were synthesized from Invitrogen, and the reaction was performed using the SuperReal PreMix Plus (SYBR Green, TIANGEN). For mature miRNA analyses, qPCR was performed using the miRcute Plus miRNA qPCR Kit (SYBR Green), and commercially available primers (TIANGEN). mRNA and miRNA expression were normalized using detection of GAPDH and U6, respectively. Each sample was run in triplicate to minimize pipetting errors. All of the primers used are listed in Supplementary Table S6.

### Protein stability assay

To assess protein stability, cells were treated with cycloheximide (20 μM) for the indicated times (2, 4, or 6 h). Cell extracts were prepared, and the expression levels of the indicated proteins were detected with western blot.

### Cell proliferation assay

Cell proliferation was measured using an MTT assay (Sigma-Aldrich). Cells were seeded at a density of 1 × 10^3^ per well onto 96-well plates and cultured for 1, 2, 3, 4, or 5 days. Cells were then incubated with 20 μl of MTT (5 mg/ml) for 4 h at 37 °C, and 150 μl of a 1:1 dimethyl sulfoxide/methanol mixture was added to solubilize the crystals for 10 min at RT. The optical density was measured at 570 nm with a reference filter of 655 nm using an xMark microplate absorbance spectrophotometer (Bio-Rad).

### Colony formation assay

For the colony formation assay, cells were seeded in 6-well plates (2 × 10^3^ cells) and cultured at 37 °C with 5% CO_2_ humidified air for 14 days. The colonies were stained with 0.5% crystal violet (1 mg/ml), and the colonies containing more than 50 cells were counted. The experiment was performed in triplicate and repeated 3 times, and the average was calculated.

### Transwell and wound healing assays

Cell migration and invasion assays were performed using chambers (8 μm pore size, Corning Costar). Briefly, 2 × 10^4^ LoVo and HCT116 cells in 200 μl of serum-free RPMI 1640 medium were placed in the uncoated upper chamber (migration assay) or the 1:2 diluted Matrigel-coated (BD Biosciences) upper chamber (invasion assay). The lower chamber was filled with 600 μl of complete medium. After cells were incubated at 37 °C, cells migrated to the bottom surface of the filter membrane were stained with 0.5% crystal violet in methanol for 45 min. For scratch-wound migration assays, a scratch wound was made using a pipette tip, and the wounds were photographed under phase-contrast microscopy (Olympus) at 0, 12, and 24 h. The percentage of healed area was measured as a ratio of the occupied area to the total area using ImageJ software (NIH Image). The assays were conducted in triplicate in three independent experiments.

### Gelatin zymography

Sodium dodecyl sulfate polyacrylamide gels (10%) with 0.1% gelatin (Solarbo) were used to identify proteins with gelatinolytic activity present in serum-free conditioned media from stable LoVo and HCT116 cells. Equally extracted proteins (40 μg) were determined by the Bradford method, mixed with sample buffer, and loaded onto the gels. After electrophoresis under nonreducing conditions at 120 V for 2 h, the gels were renatured for 1 h and then incubated at 37 °C for 16 h in digestive buffer, stained with 0.5% Coomassie blue R-250, and destained for 6–24 h until clear white band appeared on blue background. MMP-9 activity reflected by the white band was quantified by ImageJ software (NIH Image).

### Tube formation assays

Matrigel (BD Biosciences) was polymerized in a 48-well plates for 30 min at 37 °C. Stable LoVo and HCT116 cells (2 × 10^4^) were suspended in conditioned medium and seeded on growth factor-reduced Matrigel. After 12 h of incubation, tube-forming structures were analyzed by counting the number of connecting branches.

### Phospho-kinase antibody array

Human Phospho-Kinase Array Kit (R&D Systems) was performed according to the manufacturer’s instructions. The lysates of stable LoVo cells were diluted and mixed with a cocktail of biotinylated detection antibodies and incubated with the array. Then streptavidin-horseradish peroxidase and chemiluminescent detection reagents were added, and chemiluminescence was detected using image analyzer ImageQuant LAS 500 (GE Healthcare).

### In situ hybridization

We performed in situ hybridization using locked nucleic acid (LNA)-modified DNA, which was 5′- and 3′-digoxigenin labeled. The probe was used for the detection of miR-185, U6 snRNA as the positive control, and a scramble LNA probe as the negative control. All probes were obtained from Exiqon. Hybridizations were performed on 4 µm FFPE sections of tissue with primary or distant metastases of CRC and matched paracarcinomas, following the manufacturer’s protocol, with amendments as follows: proteinase K (40 µg/ml) digestion for 30 min, 40 nM of hybridization probe, a hybridization temperature of 64 °C (Tm-30 °C), and an overnight incubation with TSA/Cy3 (Perkin Elmer) solution. For the cellular staining, an anti-DC-SIGN antibody was incubated with the blocked cells for 2 h. Cells were then incubated with a secondary antibody conjugated to FITC (ZSGB-Bio) for 1 h and mounted using DAPI (Vector Laboratories). Fluorescence signals were detected and visualized at RT using a Leica TCS SP5II scanning confocal microscope (Leica). The tissues were scored by quantifying the fluorescence density of 10 separate fields in the areas with the highest intensity of fluorescence.

### Luciferase reporter assay

The DC-SIGN 3′-UTR was amplified by PCR and cloned into a pmirGLO Dual-Luciferase miRNA Target Expression Vector (Promega). The miR-185 mimics or negative controls and the pmirGLO Dual-Luciferase 3′-UTR vector were cotransfected into either HEK293 or LoVo cells using Lipofectamine 2000 (Invitrogen). Cells were harvested and lysed at 48 h posttransfection. The interactions between miR-185 and DC-SIGN 3′-UTR were measured using a Dual-Luciferase Reporter Assay System (Promega) in triplicate. The firefly luciferase activity was normalized to that of *Renilla* luciferase for each sample.

For TOPflash luciferase assay, 2 × 10^5^ stable LoVo cells infected with Lenti-DC-SIGN shRNA or control were plated into one well from 24-well plates and then transfected with 50 ng of the TCF optimal (TOP) or mutant negative control (FOP) luciferase reporter, 10 ng of the pRL-TK vector (Promega), and 50 ng of the expression vector by using Lipofectamine 2000. Cells were transfected as described above, and treated with DC-SIGN mAb and/or LY294002. After 24 h, cells were lysed, and the luciferase activities were detected and analyzed as described above.

### Chromatin immunoprecipitation assay

Chromatin immunoprecipitation was performed using an EZ-Magna ChIP Assay Kit (Millipore) according to manufacturer’s protocol. Briefly, cells were cross-linked with 1% formaldehyde for 10 min. Cells were then washed with cold PBS, scraped, and collected on ice. Next, cells were harvested, lysed, and sonicated. After centrifugation, the supernatant was collected, and an equal amount of sonicated DNA fragments was immunoprecipitated with antibodies against TCF1, LEF1, or nonspecific IgG (Santa Cruz and Cell Signaling) at 4 °C overnight. The antibody-protein-DNA complexes were isolated by immunoprecipitation with protein A/G magnetic beads. Following extensive washing, bound DNA fragments were eluted and amplified by PCR.

### Mouse xenograft and metastasis model

BALB/c athymic nude mice that were 4–6 weeks of age were purchased from the Animal Center of Dalian Medical University (Dalian, China). To evaluate the in vivo tumorigenic effects, LoVo cells (2 × 10^6^ cells in 0.2 ml PBS per mouse) or HCT116 cells (5 × 10^6^ cells in 0.2 ml PBS per mouse) transfected with control or DC-SIGN shRNA were subcutaneously injected into the flanks of the nude mice. The caliper measurements were performed every 4 days and the tumor volume (*V*) was calculated using the formula *V* = (*L* × *W*^2^)/2, where *L* is the longest diameter, and *W* is the diameter perpendicular to *L* of the tumor. The tumor volumes of the mice were recorded for 44 days, after which the mice were euthanized.

The capacity for metastasis to the liver was determined using a previously described method. Briefly, 5 × 10^6^ of HCT116 or LoVo cells that were transfected with lentiviral vectors were injected into the spleens of the recipient mice. The animals were killed after 5–6 weeks, and the spleens, livers, lungs, and lymph nodes were dissected and embedded in paraffin. The mean volumes of the tumors and the numbers of metastases were calculated. The dissected tumors were collected and prepared for RNA and protein isolation and IHC staining. All of the animal experiments were approved by the Animal Center of Dalian Medical University in accordance with the national guidelines for the care and use of laboratory animals.

### Fluorescence imaging and analysis

The mice were anesthetized, and images were taken with the mice in a supine position using the in vivo Imaging System Fx Pro coupled with living Image acquisition and analysis software (Kodak Inc.). With the aid of fluorescence imaging techniques, the GFP protein can locate the tumor region more precisely, allowing the growth of the tumor to be monitored. For the FRI plots, photon flux was calculated for each mouse by using a rectangular region of interest encompassing the abdomen and thorax of the mouse.

### Statistical analysis

All experiments were repeated for at least three times, and the data are presented as mean ± SD. The statistical analyses were performed using SPSS software 17.0 and GraphPad Prism 5.0. The graphs were analyzed by either ANOVA (multiple groups) or *t* tests (two groups). A *P* value of 0.05 or less was considered as statistically significant.

## Supplementary information

Supplementary figures legends

Supplementary figures

Supplementary tables
