# Correction to: DC-SIGN–LEF1/TCF1–miR-185 feedback loop promotes colorectal cancer invasion and metastasis

**DOI:** 10.1038/s41418-021-00736-9

**Published:** 2021-03-04

**Authors:** Menglang Yuan, Xinsheng Zhang, Jingbo Zhang, Keyong Wang, Yu Zhang, Wei Shang, Yinan Zhang, Jingyi Cui, Xiaomeng Shi, Heya Na, Deyu Fang, Yunfei Zuo, Shuangyi Ren

**Affiliations:** 1 Department of General Surgery, The Second Affiliated Hospital of Dalian Medical University, 116023 Dalian, China; 2 Department of Clinical Biochemistry, College of Laboratory Diagnostic Medicine, Dalian Medical University, 116044 Dalian, China; 3 Department of Pathology, Northwestern University Feinberg School of Medicine, Chicago, IL 60611 USA

**Keywords:** Metastasis, Oncogenes

Correction to: *Cell Death & Differentiation*

10.1038/s41418-019-0361-2

The original version of this article unfortunately contained a mistake. Since online publication of this article, the authors noticed that there was an error in Supplementary Figure 7b. An incorrect image was used in invasion panel for DC-SIGN+miR-185. The image has been updated online.

## Supplementary information

Supplementary Figures

