# Supplementary material for: DC-SIGN–LEF1/TCF1–miR-185 feedback loop promotes colorectal cancer invasion and metastasis
Source: Cell Death Differ. 2019 Jun 19;27(1):379–95. doi: 10.1038/s41418-019-0361-2 (PMC7205996; doi:10.1038/s41418-019-0361-2)
Supplement: Supplementary file 1 — Supplementary figures legends [file 41418_2019_361_MOESM1_ESM.docx]

**Supplementary Figure Legends**

**Supplementary Figure S1. Higher soluble DC-SIGN levels are significantly associated with metastatic CRC.** **a** ROC curves revealed that DC-SIGN had good diagnostic accuracy for metastatic CRC. The optimal cut-off value for sDC-SIGN was 0.3004 μg/ml. **b** The positive rate of DC-SIGN was analyzed in patients with primary tumors or metastasis CRC, and DC-SIGN by CEA status in patients with mCRC. **c** Two-parameter (CEA or DC-SIGN in serum) was used to discriminate between primary and metastasis in CRC. The cut-off value was 0.3004 μg/mL for DC-SIGN, and 5.0 ng/mL for CEA. The corresponding cut-off values are marked by gray lines.

**Supplementary Figure S2. Genomic alterations of *DC-SIGN* in 276 CRC cases from TCGA database.** **a** Genomic alterations of *DC-SIGN* were analyzed in 276 colorectal cancer cases from TCGA database. **b** The effect of *DC-SIGN* mutations on DC-SIGN expression and DC-SIGN/Lyn interaction was examined in LoVo cells by immunoprecipitation assay and western blot.

**Supplementary Figure S3. Silencing of DC-SIGN inhibits proliferation and migration of CRC cells.** **a** A schematic of the lentiviral vector used for cloning the shRNA. **b** The infection efficiency of the lentivirus in LoVo and HCT116 cells. Because this vector contains a GFP fragment, the cells emit green fluorescence when infected by the lentivirus. **c** The effect of DC-SIGN-specific shRNA on the protein levels in human colon cancer cell lines is detected by western blot. **d, e** The effect of DC-SIGN-specific shRNA on the proliferation and migration of human colon cancer cells by MTT (**d**) and wound healing (**e**) assays, respectively. Images shown are representative of three independent experiments. Data, mean ± SD. **P* < 0.05, **, *P* < 0.01.

**Supplementary Figure S4. The metastasized liver and lung tumors from LoVo cells resembled the primary tumor.** LoVo cells stably expressing either control (*left panel*) or DC-SIGN shRNA (*right panel*) were injected subcutaneously into the flank or spleen of each nude mouse. The tissues were stained with colon cancer markers CK7, CK20, CDX2, or proliferation marker Ki-67.

**Supplementary Figure S5. DC-SIGN is a direct target of miR-185 in CRC cells. a** A comparison of the nucleotide sequences of the miR-185 seed sequences with putative target sequences in different species. **b** Secondary structure of the specific miR-185-binding site and its flank regions in the DC-SIGN 3’-UTR. **c** Correlation between DC-SIGN mRNA and miR-185 levels in colon cancer cells. **d** Lentiviral vectors with miR-185 or miR-185 inhibitor were infected into LoVo and HCT116 cells, followed by transfection of DC-SIGN vector or Lenti-DC-SIGN shRNA. Then total RNA was extracted and applied to real-time PCR. Cell lysates were applied to Western blot. Data, mean ± SD. **, *P* < 0.01.

**Supplementary Figure S6. The miR-185/DC-SIGN axis regulates the migration and proliferation of CRC cells. a, b** Lentiviral vectors with miR-185 or miR-185 inhibitor were infected into LoVo or HCT116 cells, followed by transfection of DC-SIGN vector (**a**) or DC-SIGN shRNA (**b**). Then cells were applied to wound-healing analysis. **c** CRC cells proliferation were assessed by colony formation analysis. Images shown are representative of three independent experiments. **d** CRC cells proliferation were assessed by MTT analysis. Data, mean ± SD. **, *P* < 0.01.

**Supplementary Figure S7. miR-185 represses DC-SIGN to suppress CRC invasion and migration. a** LoVo cells that were co-transfected miR-185 with both a DC-SIGN wild or mutant type vector, and cell lysates were applied to Western blot. **b** Cells were transfected as indicated, and applied to transwell analysis. Images shown are representative of three independent experiments. Data, mean ± SD. **P* < 0.05, **, *P* < 0.01.

**Supplementary Figure S8. ERK signaling is not involved in DC-SIGN activation-induced metastasis in CRC. a** LoVo cells were treated as indicated, and the lysates were probed for phosphotyrosine residues by western blot. **b** LoVo cells were treated with DC-SIGN agonistic antibody (B-2, 10 μg/ml) for 15 minutes and/or ERK inhibitor (U1026, 50 μM) for 24 hours, and applied to western blot analysis.

**Supplementary Figure S9. The effect of Lyn on DC-SIGN expression in CRC cells. a** The expression profiles of Lyn mRNA in colon cancer cells. **b** LoVo and HCT116 cells were transiently transfected with scramble siRNA (Scr siRNA) or Lyn siRNA, LS174T cells were transiently transfected with vector or Flag-Lyn, and applied to real-time PCR assay. **c** LoVo cells serum-starved for 24 hours were either treated or not treated with DC-SIGN agonistic antibody (B-2, 10 μg/ml) for 15 minutes, then lysed and immunoprecipitated with DC-SIGN or Lyn antibody and immunoblotted with the indicated antibodies. **d** The lysates of LoVo cells were applied to immunoprecipitation using Lyn antibody. The immunoprecipitates were examined to blot Akt, GSK3β, β-catenin, LEF1 and TCF1, as well as the metastases-related protein MMP-9 and VEGF. Data, mean ± SD. N.S., nonsignificant.

**Supplementary Figure S10. TCF1/LEF1 does not bind to the miR-185 promoter in the intestinal epithelial cells.** Chromatin of HIEC cells cultured in the absence and presence of DC-SIGN mAb was subjected to chromatin immunoprecipitation with antibodies against LEF1 or TCF1, followed by real-time PCR analysis. The results are the average of three independent experiments. Data, mean ± SD. N.S., nonsignificant.
