# Supplementary material for: DC-SIGN–LEF1/TCF1–miR-185 feedback loop promotes colorectal cancer invasion and metastasis
Source: Cell Death Differ. 2019 Jun 19;27(1):379–95. doi: 10.1038/s41418-019-0361-2 (PMC7205996; doi:10.1038/s41418-019-0361-2)
Supplement: Supplementary file 2 — Supplementary figures [file 41418_2019_361_MOESM2_ESM.pdf]

Figure S1

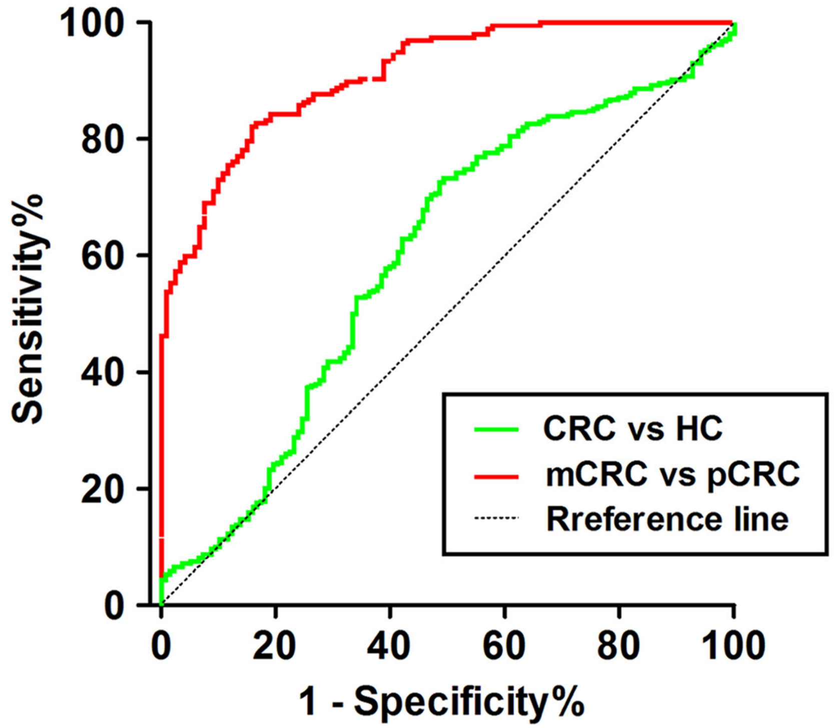

Contrast	AUC	SE	95% CI	P value
mCRC vs pCRC	0.9106	0.0153	0.8805-0.9407	$<$0.0001
CRC vs HC	0.5993	0.0298	0.5409-0.6578	0.0007

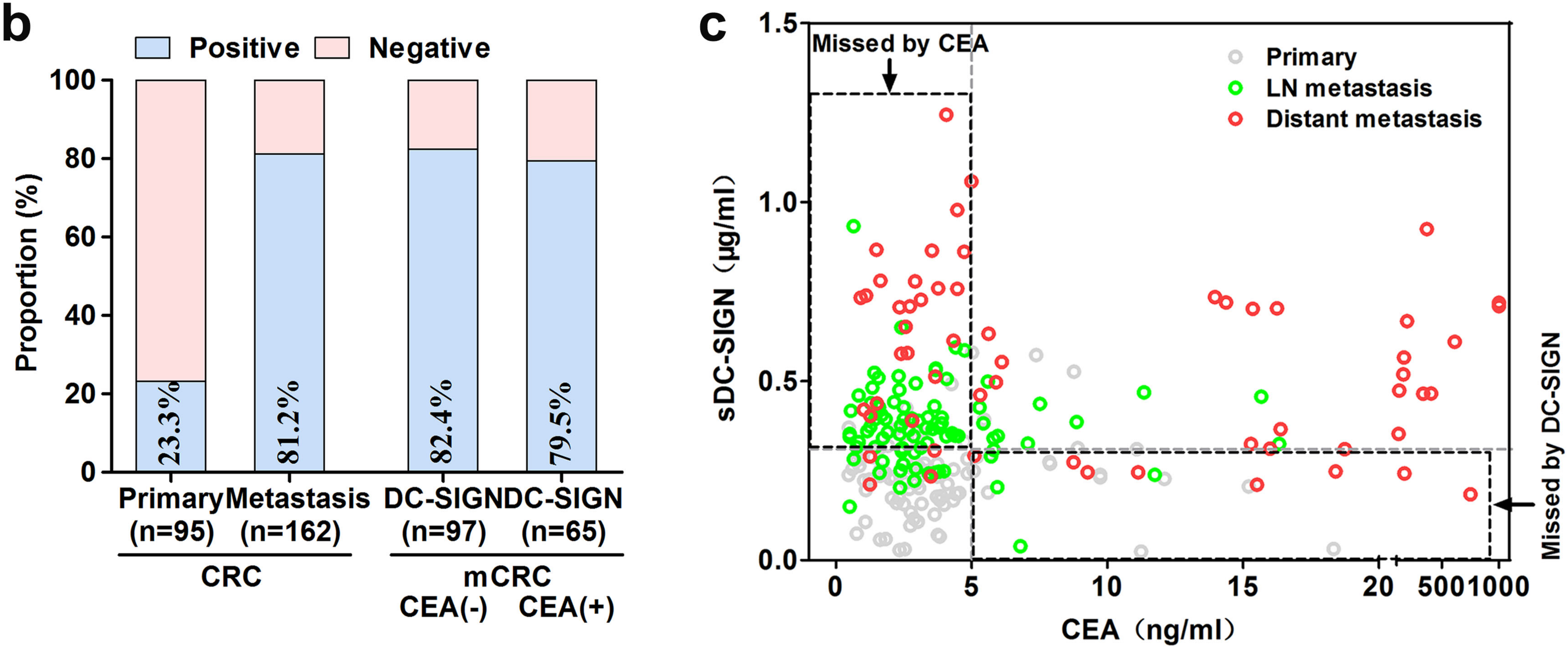

Figure S2

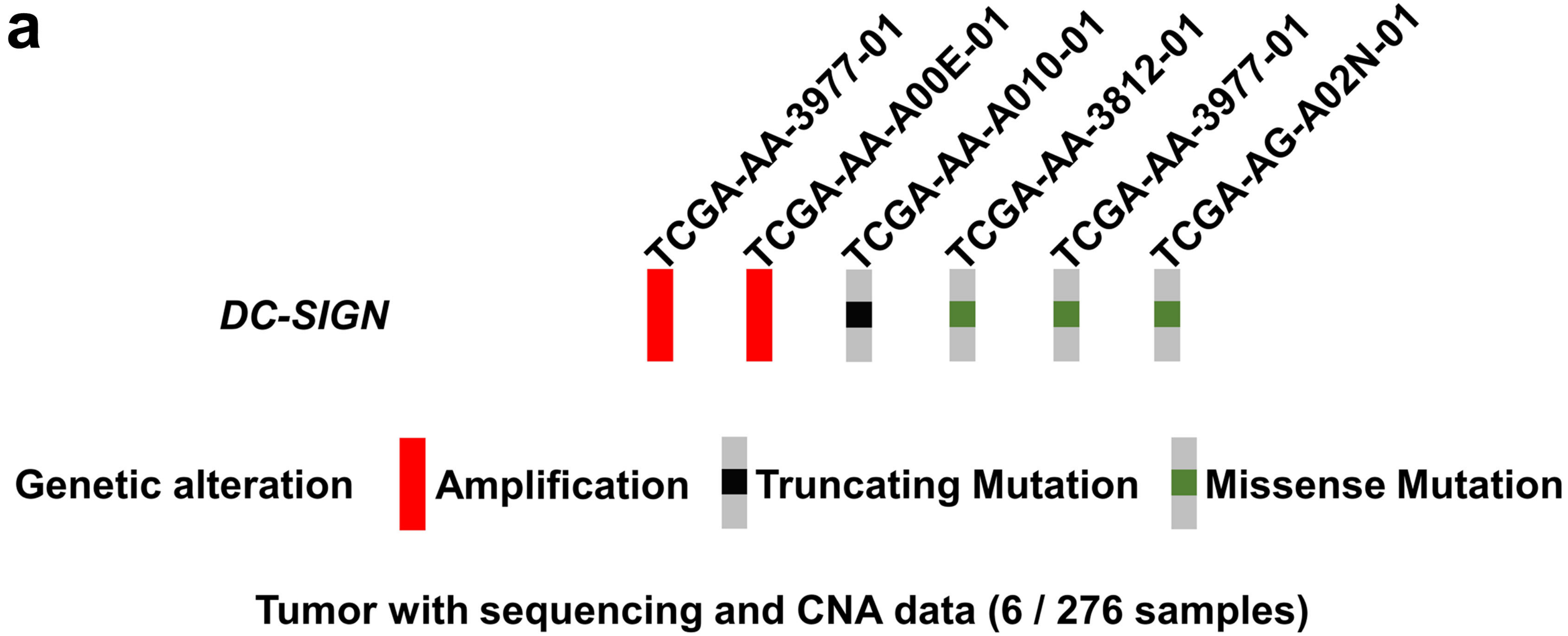

a
TCGA-AA-3977-01
TCGA-AA-A00E-01
TCGA-AA-A010-01
TCGA-AA-3812-01
TCGA-AA-3977-01
TCGA-AG-A02N-01
DC-SIGN
Genetic alteration
Amplification
Truncating Mutation
Missense Mutation
Tumor with sequencing and CNA data (6 / 276 samples)

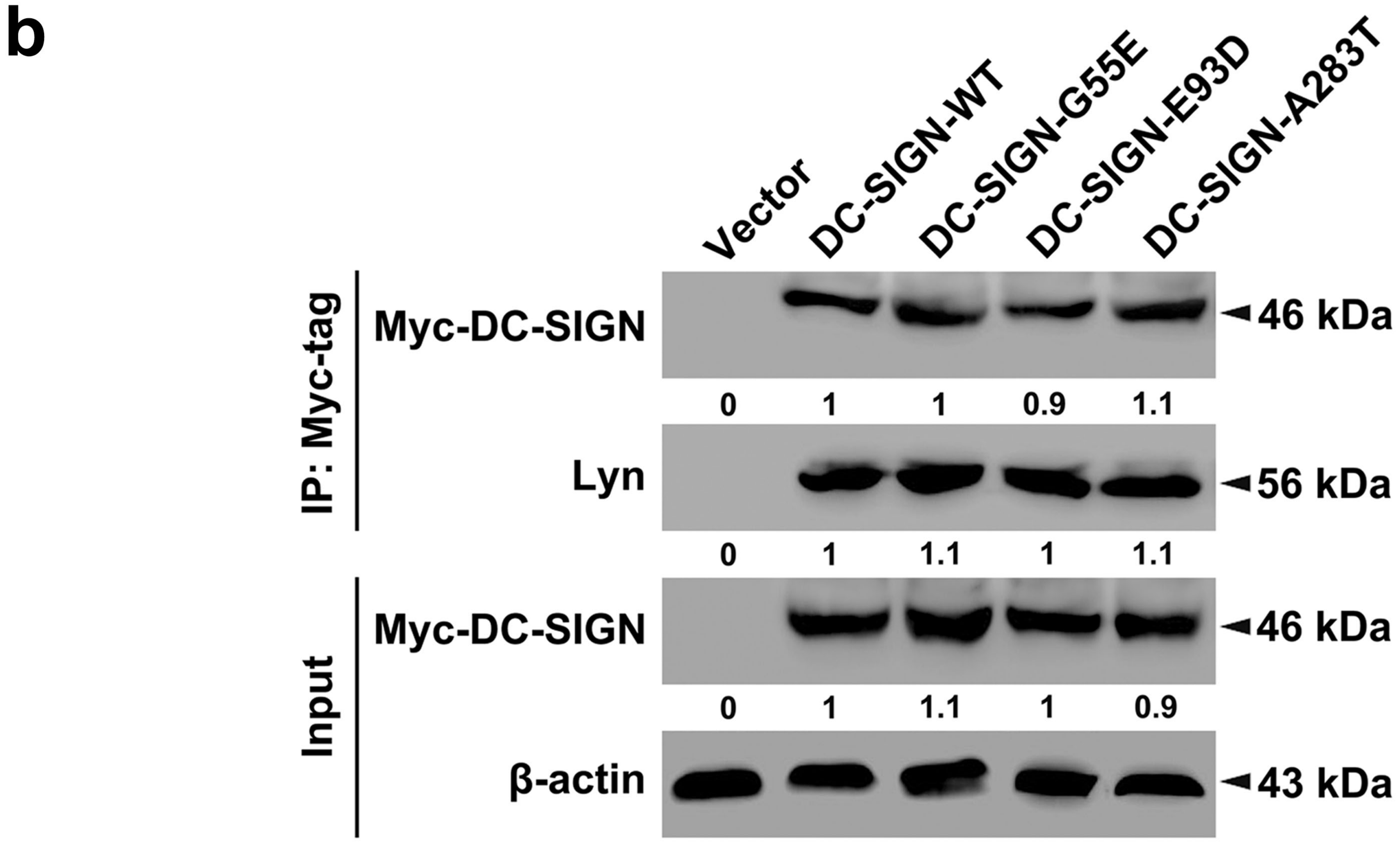

b
Vector
DC-SIGN-WT
DC-SIGN-G55E
DC-SIGN-E93D
DC-SIGN-A283T
IP: Myc-tag
Myc-DC-SIGN
46 kDa
0 1 1 0.9 1.1
Lyn
56 kDa
0 1 1.1 1 1.1
Input
Myc-DC-SIGN
46 kDa
0 1 1.1 1 0.9
β-actin
43 kDa

Figure S3

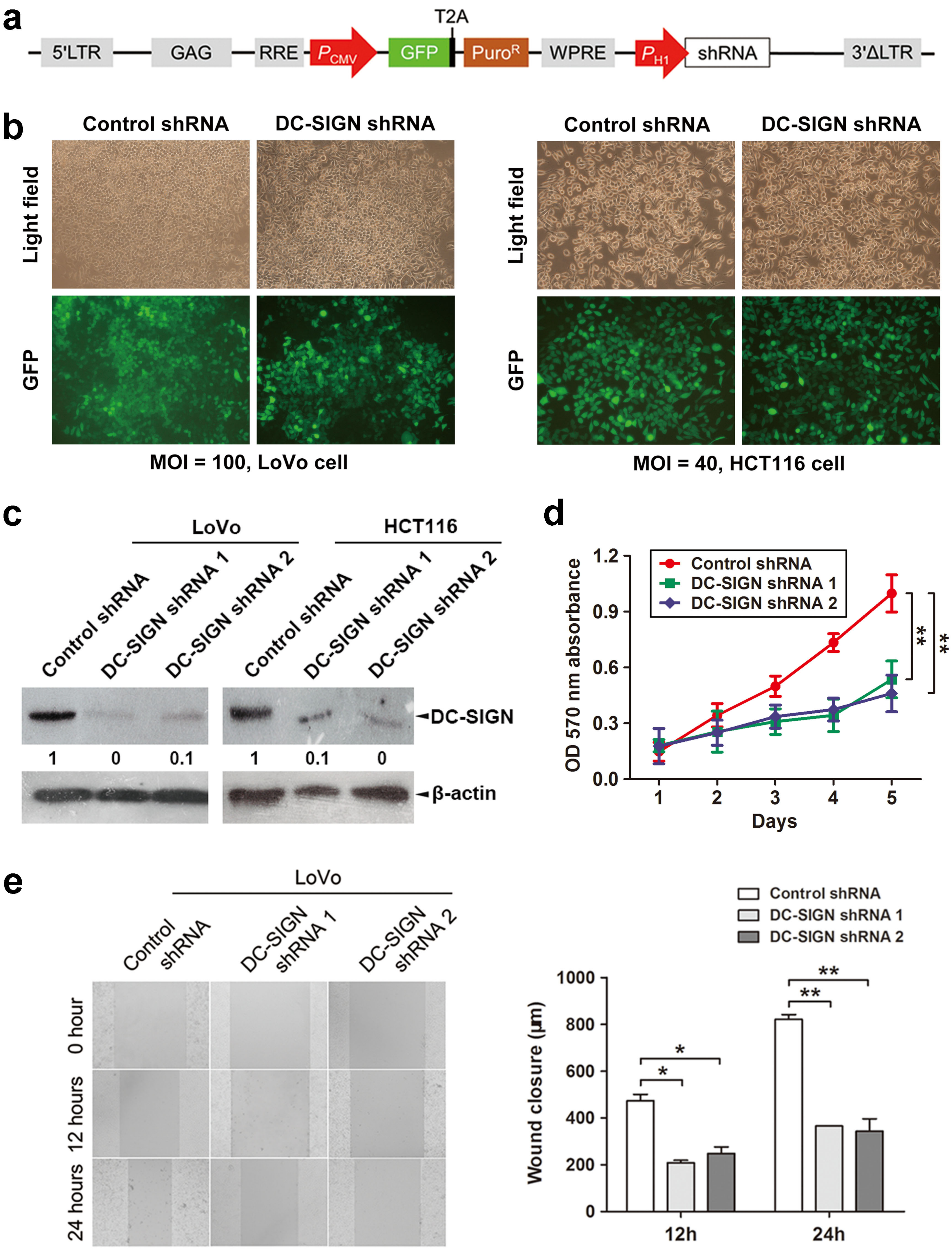

a
T2A
5'LTR
GAG
RRE
P_{CMV}
GFP
Puro^R
WPRE
P_{H1}
shRNA
3'ΔLTR
b
Control shRNA
DC-SIGN shRNA
Light field
GFP
MOI = 100, LoVo cell
MOI = 40, HCT116 cell
c
LoVo
HCT116
Control shRNA
DC-SIGN shRNA 1
DC-SIGN shRNA 2
DC-SIGN
1
0
0.1
1
0.1
0
β-actin
d
Control shRNA
DC-SIGN shRNA 1
DC-SIGN shRNA 2
OD 570 nm absorbance
Days
**
**
e
LoVo
Control shRNA
DC-SIGN shRNA 1
DC-SIGN shRNA 2
0 hour
12 hours
24 hours
Wound closure (μm)
12h
24h
*
*
**
**

Figure S4

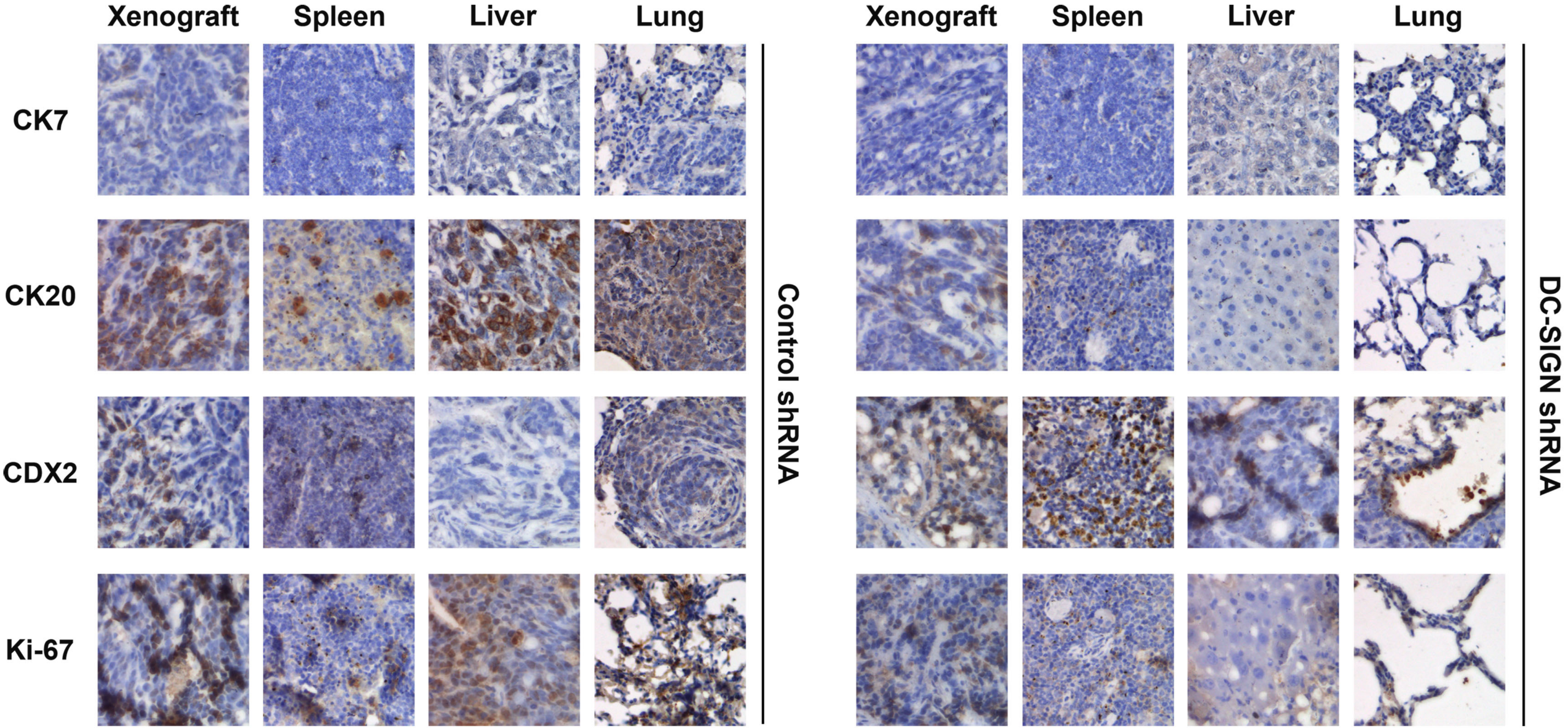

Xenograft
Spleen
Liver
Lung
Xenograft
Spleen
Liver
Lung
CK7
CK20
CDX2
Ki-67
Control shRNA
DC-SIGN shRNA

Figure S5

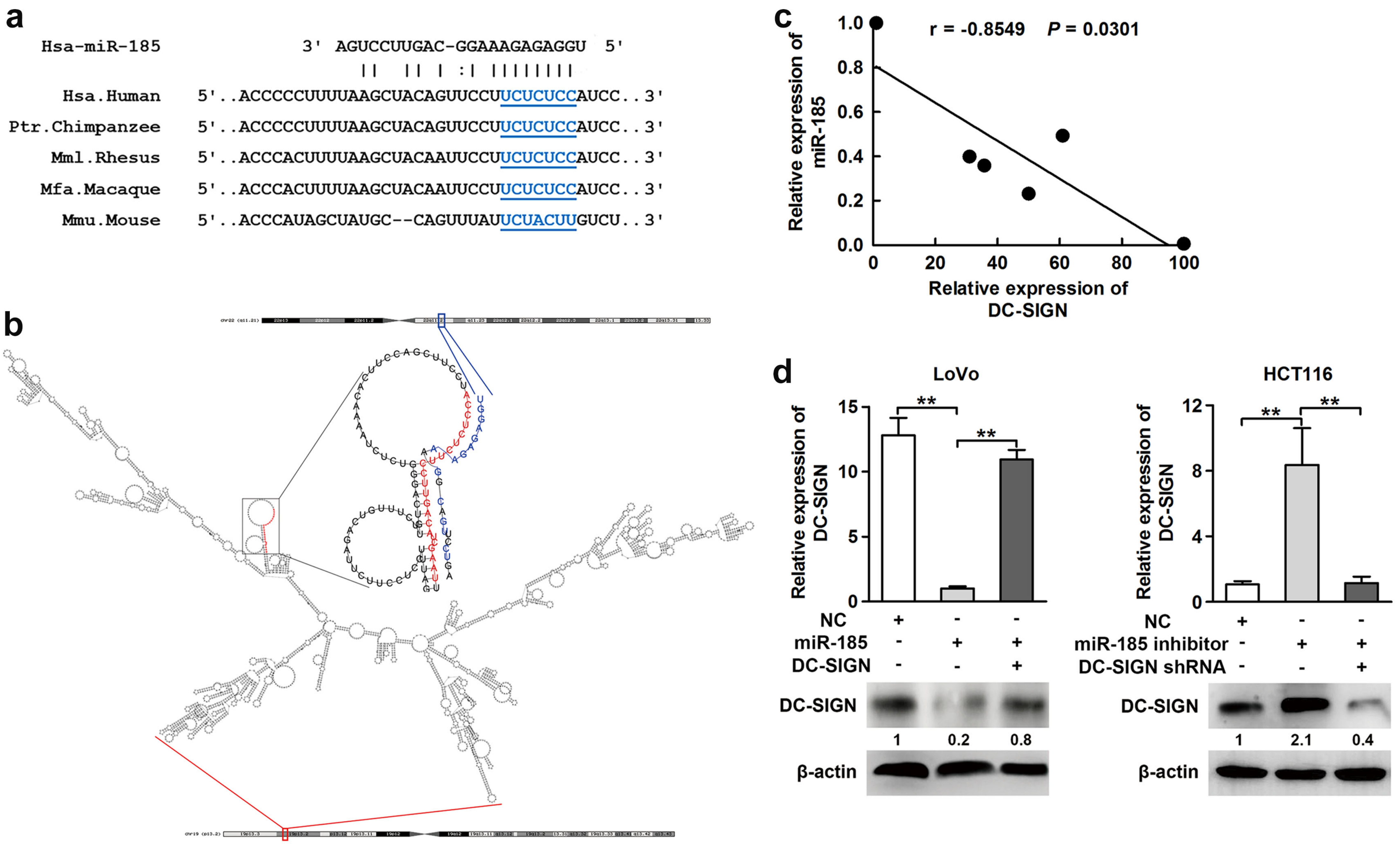

a
Hsa-miR-185 3' AGUCCUUGAC-GGAAAGAGAGGU 5'
Hsa.Human 5'..ACCCCCUUUUAAGCUACAGUUCCUUCUCUCCAUCC..3'
Ptr.Chimpanzee 5'..ACCCCCUUUUAAGCUACAGUUCCUUCUCUCCAUCC..3'
Mml.Rhesus 5'..ACCCACUUUUAAGCUACAAUUCCUUCUCUCCAUCC..3'
Mfa.Macaque 5'..ACCCACUUUUAAGCUACAAUUCCUUCUCUCCAUCC..3'
Mmu.Mouse 5'..ACCCAUAGCUAUGC--CAGUUUAUUCUACUUGUCU..3'
b
c
r = -0.8549 P = 0.0301
Relative expression of miR-185
Relative expression of DC-SIGN
1.0
0.8
0.6
0.4
0.2
0.0
0
20
40
60
80
100
d
LoVo
HCT116
Relative expression of DC-SIGN
15
10
5
0
12
8
4
0
**
NC + - -
miR-185 - + +
DC-SIGN - - +
NC + - -
miR-185 inhibitor - + +
DC-SIGN shRNA - - +
DC-SIGN
1 0.2 0.8
1 2.1 0.4
β-actin

Figure S6

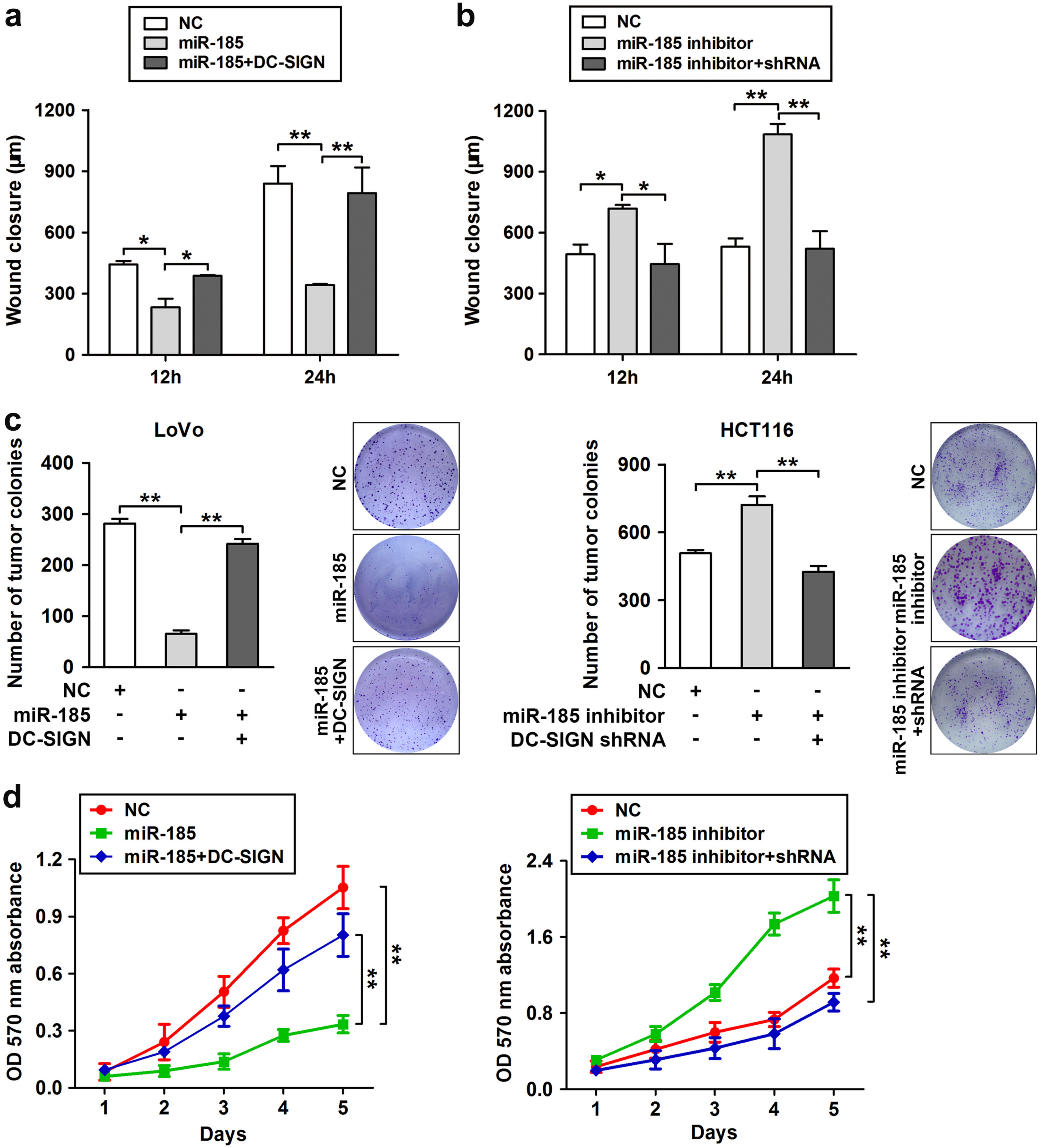
a
NC
miR-185
miR-185+DC-SIGN
Wound closure (μm)
1200
900
600
300
0
12h
24h
*
**
b
NC
miR-185 inhibitor
miR-185 inhibitor+shRNA
Wound closure (μm)
12h
24h
c
LoVo
Number of tumor colonies
400
300
200
100
0
NC
miR-185
DC-SIGN
+
-
miR-185
+DC-SIGN
HCT116
Number of tumor colonies
900
600
300
0
miR-185 inhibitor
DC-SIGN shRNA
miR-185 inhibitor
miR-185 inhibitor
+shRNA
d
NC
miR-185
miR-185+DC-SIGN
OD 570 nm absorbance
1.2
0.9
0.6
0.3
0.0
Days
1
2
3
4
5
NC
miR-185 inhibitor
miR-185 inhibitor+shRNA
2.4
1.6
0.8
0.0
Days

Figure S7

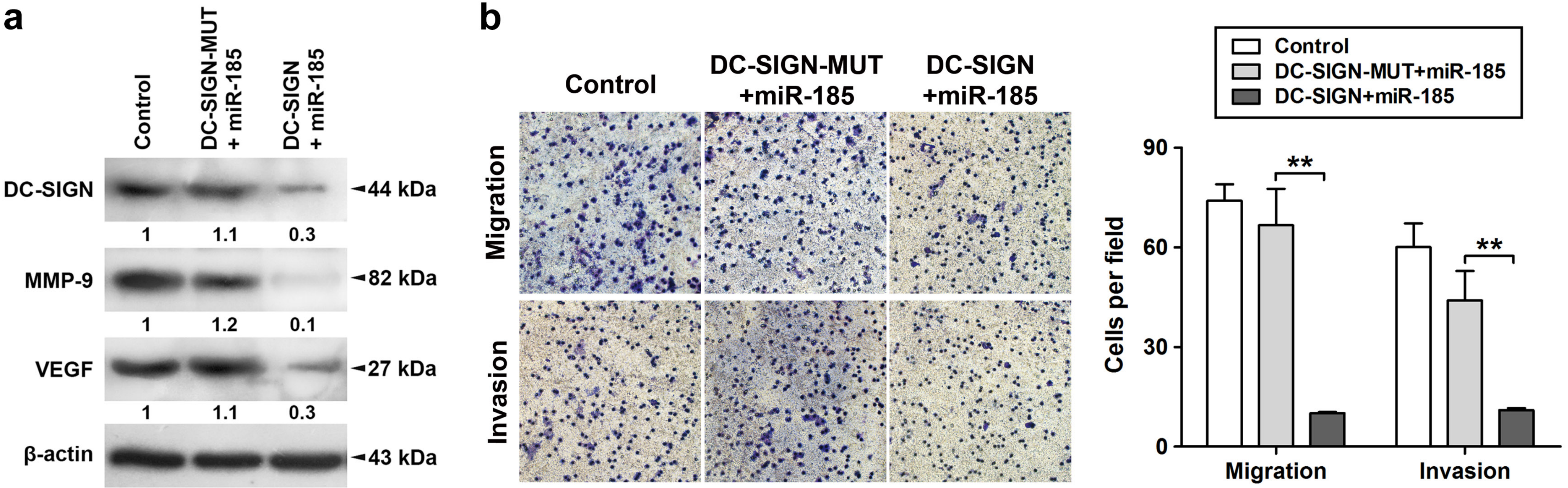
a
Control
DC-SIGN-MUT + miR-185
DC-SIGN + miR-185
DC-SIGN
◄44 kDa
1
1.1
0.3
MMP-9
◄82 kDa
1
1.2
0.1
VEGF
◄27 kDa
1
1.1
0.3
β-actin
◄43 kDa
b
Control
DC-SIGN-MUT +miR-185
DC-SIGN +miR-185
Migration
Invasion
Control
DC-SIGN-MUT+miR-185
DC-SIGN+miR-185
Cells per field
90
60
30
0
**
**
Migration
Invasion

Figure S8

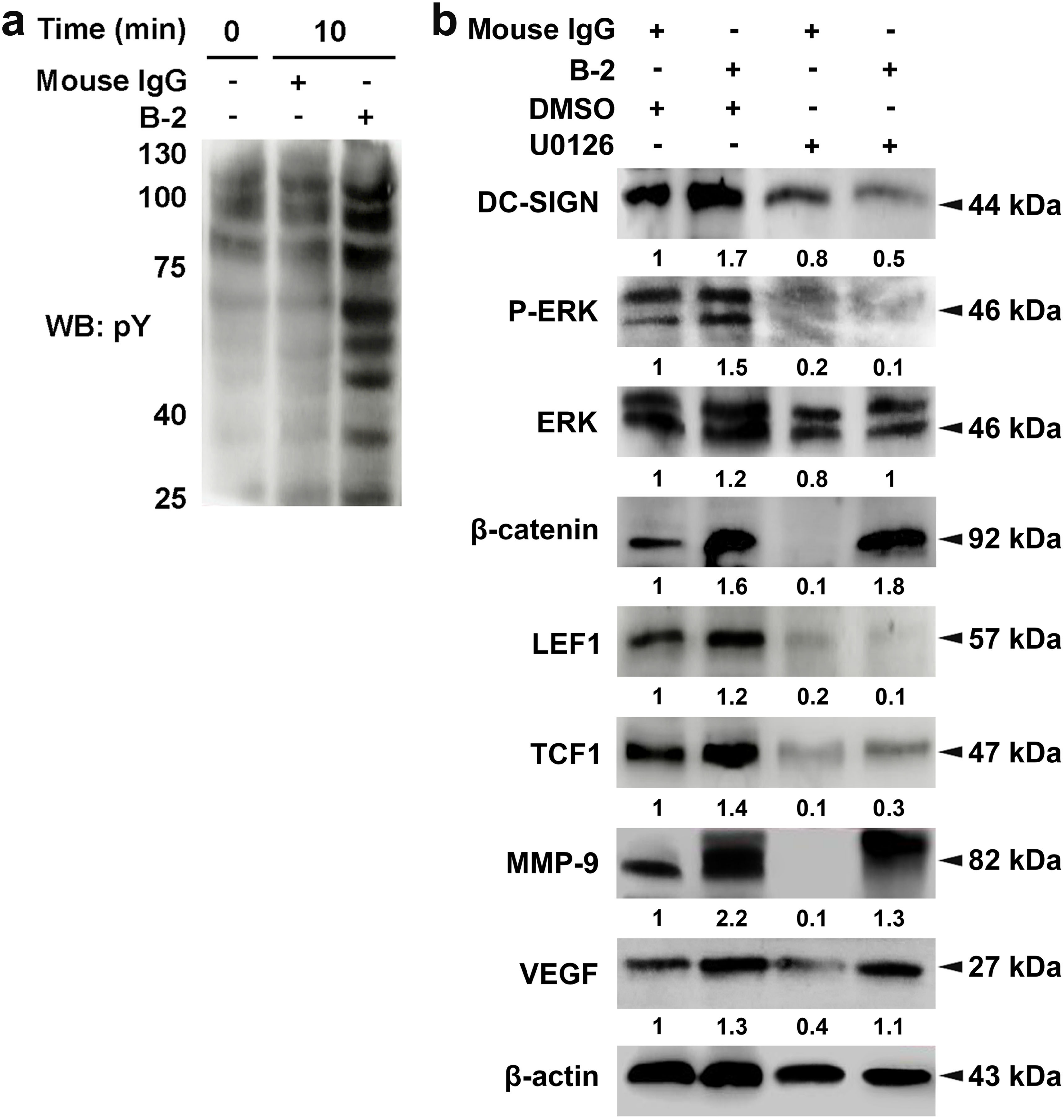
a
Time (min)
0
10
Mouse IgG
B-2
130
100
75
40
25
WB: pY
b
Mouse IgG
B-2
DMSO
U0126
DC-SIGN
44 kDa
1 1.7 0.8 0.5
P-ERK
46 kDa
1 1.5 0.2 0.1
ERK
46 kDa
1 1.2 0.8 1
β-catenin
92 kDa
1 1.6 0.1 1.8
LEF1
57 kDa
1 1.2 0.2 0.1
TCF1
47 kDa
1 1.4 0.1 0.3
MMP-9
82 kDa
1 2.2 0.1 1.3
VEGF
27 kDa
1 1.3 0.4 1.1
β-actin
43 kDa

Figure S9

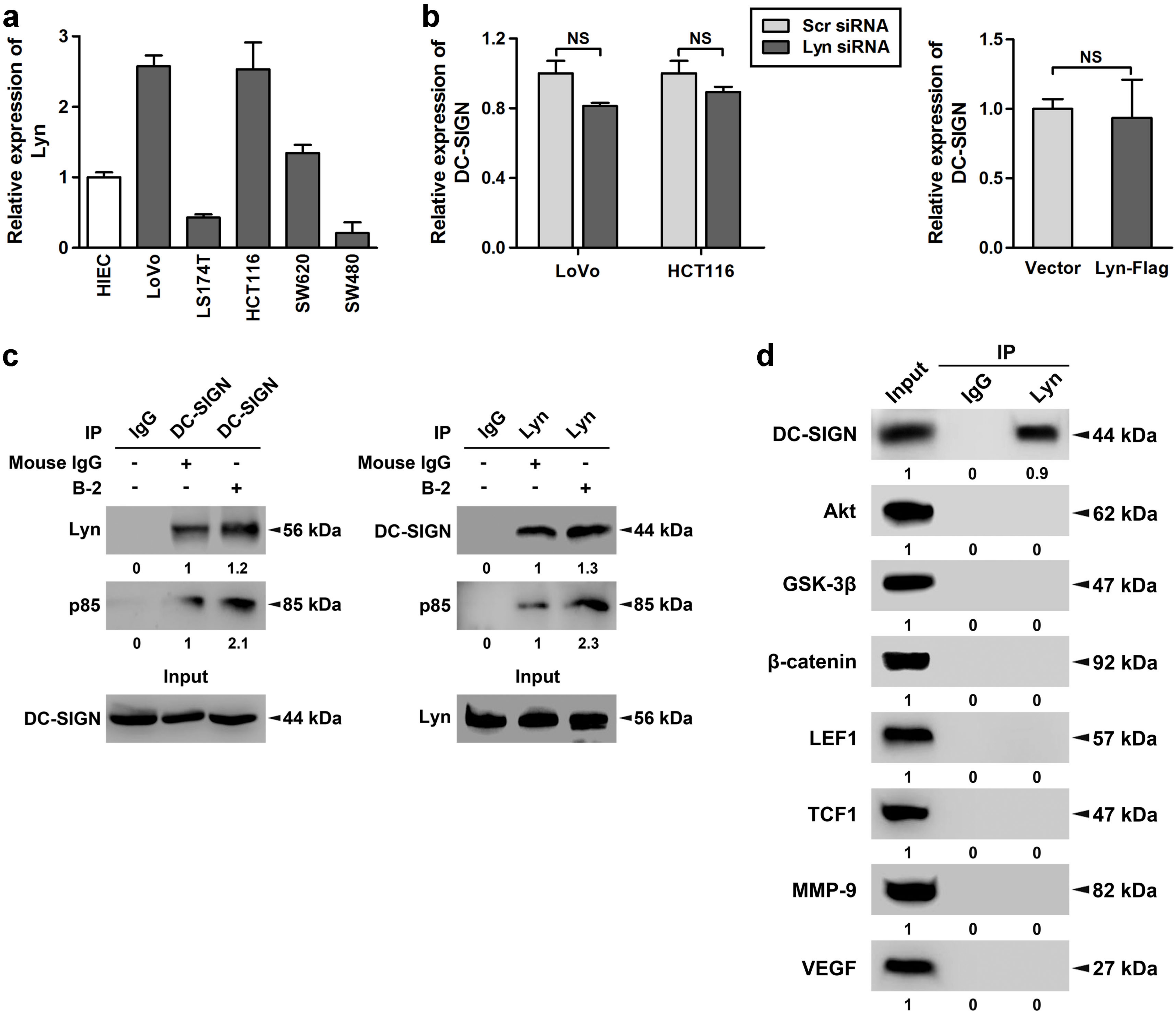
a
Relative expression of Lyn
3
2
1
0
HIEC
LoVo
LS174T
HCT116
SW620
SW480
b
Relative expression of DC-SIGN
1.2
0.8
0.4
0.0
NS
NS
Scr siRNA
Lyn siRNA
LoVo
HCT116
1.5
1.0
0.5
0.0
NS
Vector
Lyn-Flag
c
IP
IgG
DC-SIGN
DC-SIGN
Mouse IgG - + -
B-2 - - +
Lyn
56 kDa
0 1 1.2
p85
85 kDa
0 1 2.1
Input
DC-SIGN
44 kDa
IP
IgG
Lyn
Lyn
Mouse IgG - + -
B-2 - - +
DC-SIGN
44 kDa
0 1 1.3
p85
85 kDa
0 1 2.3
Input
Lyn
56 kDa
d
IP
Input
IgG
Lyn
DC-SIGN
44 kDa
1 0 0.9
Akt
62 kDa
1 0 0
GSK-3β
47 kDa
1 0 0
β-catenin
92 kDa
1 0 0
LEF1
57 kDa
1 0 0
TCF1
47 kDa
1 0 0
MMP-9
82 kDa
1 0 0
VEGF
27 kDa
1 0 0

Figure S10

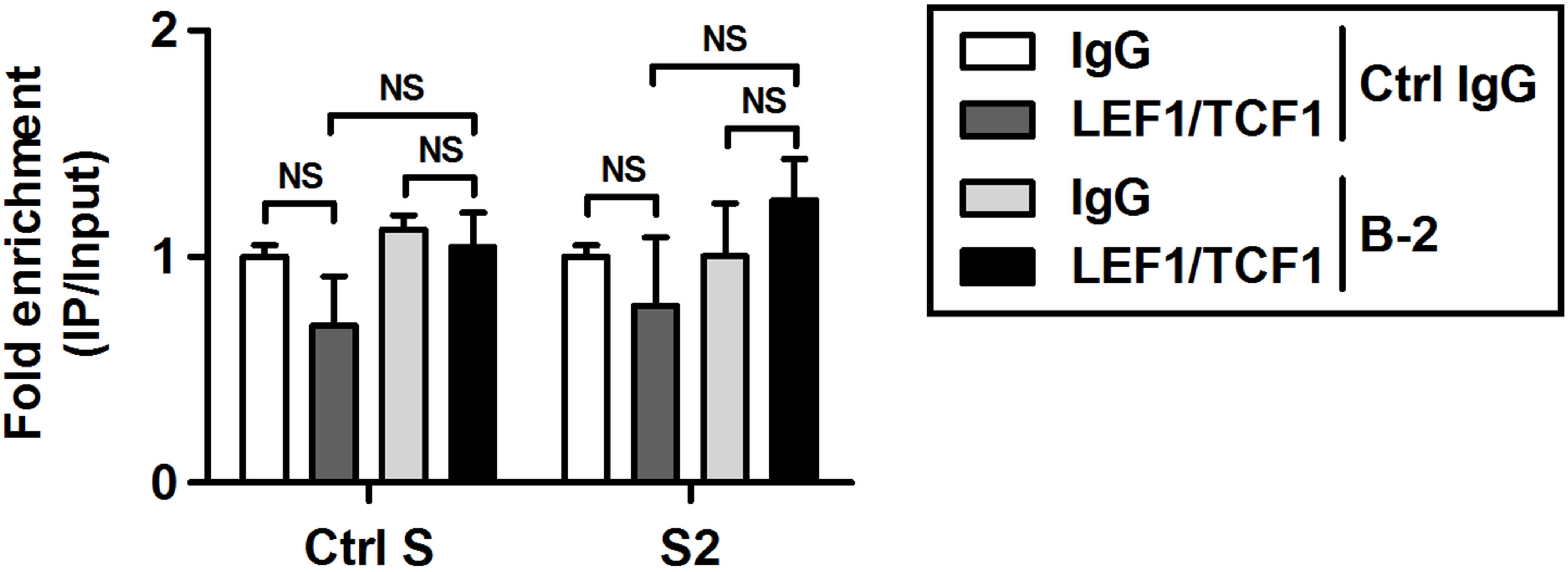

Fold enrichment
(IP/Input)
2
1
0
NS
NS
NS
NS
NS
NS
Ctrl S
S2
IgG
LEF1/TCF1
Ctrl IgG
IgG
LEF1/TCF1
B-2
