# Supplementary material for: DC-SIGN–LEF1/TCF1–miR-185 feedback loop promotes colorectal cancer invasion and metastasis
Source: Cell Death Differ. 2019 Jun 19;27(1):379–95. doi: 10.1038/s41418-019-0361-2 (PMC7205996; doi:10.1038/s41418-019-0361-2)
Supplement: Supplementary file 3 — Supplementary tables [file 41418_2019_361_MOESM3_ESM.docx]

**Supplementary Table S1.** Clinical significance of tissue and serum DC-SIGN expression in matched tissue and serum in patients with colorectal cancer.

|  | **Serum DC-SIGN** | | |  | **Tissue DC-SIGN** | | |
| --- | --- | --- | --- | --- | --- | --- | --- |
|  | **n** | **Mean±SD** | **p Value** |  | **n** | **Mean±SD** | **p Value** |
| Gender |  |  | 0.396 |  |  |  | 0.093 |
| Male | 159 | 0.367±0.195 |  |  | 34 | 0.318±0.072 |  |
| Female | 129 | 0.387±0.214 |  |  | 22 | 0.285±0.067 |  |
| Age (years) |  |  | 0.801 |  |  |  | 0.933 |
| < 60 | 119 | 0.379±0.201 |  |  | 27 | 0.302±0.071 |  |
| **≥** 60 | 169 | 0.373±0.206 |  |  | 29 | 0.301±0.084 |  |
| Location of tumor |  |  | 0.049 |  |  |  | 0.317 |
| Proximal | 101 | 0.396±0.231 |  |  | 26 | 0.318±0.085 |  |
| Distal | 105 | 0.393±0.181 |  |  | 19 | 0.292±0.078 |  |
| Rectum | 82 | 0.329±0.189 |  |  | 11 | 0.280±0.047 |  |
| Tumor size (cm) |  |  | 0.070 |  |  |  | 0.388 |
| < 5 | 143 | 0.354±0.181 |  |  | 23 | 0.291±0.064 |  |
| **≥** 5 | 145 | 0.397±0.216 |  |  | 33 | 0.309±0.085 |  |
| Tumor invasion |  |  | 0.014 |  |  |  | 0.235 |
| ECC(T1、T2) | 26 | 0.282±0.256 |  |  | 4 | 0.257±0.101 |  |
| ACC(T3、T4) | 262 | 0.385±0.196 |  |  | 52 | 0.305±0.075 |  |
| Lymph node metastasis |  |  | < 0.0001 |  |  |  | 0.675 |
| Negative | 143 | 0.328±0.194 |  |  | 17 | 0.295±0.069 |  |
| Positive | 145 | 0.423±0.202 |  |  | 39 | 0.308±0.081 |  |
| Distant metastasis |  |  | < 0.0001 |  |  |  | 0.334 |
| Negative | 231 | 0.346±0.169 |  |  | 38 | 0.309±0.080 |  |
| Positive | 57 | 0.497±0.276 |  |  | 18 | 0.287±0.069 |  |
| TNM stage |  |  | < 0.0001 |  |  |  | 0.107 |
| I | 12 | 0.107±0.127 |  |  | 0 |  |  |
| II | 97 | 0.279±0.122 |  |  | 12 | 0.274±0.068 |  |
| III | 103 | 0.375±0.125 |  |  | 26 | 0.325±0.082 |  |
| IV | 76 | 0.544±0.253 |  |  | 18 | 0.287±0.069 |  |
| Vessel invasion |  |  | 0.274 |  |  |  | 0.799 |
| Negative | 216 | 0.368±0.209 |  |  | 39 | 0.301±0.080 |  |
| Positive | 72 | 0.399±0.187 |  |  | 17 | 0.306±0.072 |  |
| Number of polyps |  |  | 0.108 |  |  |  | 0.008 |
| 0-2 | 221 | 0.386±0.211 |  |  | 50 | 0.292±0.076 |  |
| **≥** 3 | 67 | 0.341±0.175 |  |  | 6 | 0.379±0.039 |  |

**Abbreviations:** ECC, early colorectal cancer; ACC, advanced colorectal cancer; TNM, tumor node metastasis; NS, not significant.

**Supplementary Table S2.** Univariate and multivariate analysis of the correlation between clinicopathological parameters and overall survival time of patients with colorectal cancer.

| **Characteristic** | **Univariate** | |  | **Multivariate** | |
| --- | --- | --- | --- | --- | --- |
|  | **HR (95% CI)** | **p Value** |  | **HR (95% CI)** | **p Value** |
| Age (**≥** 60 vs < 60 yr) | 1.384 (0.667-2.871) | 0.383 |  |  |  |
| Tumor location (rectum vs others) | 1.485 (0.713-3.093) | 0.290 |  | 0.736 (0.335-1.671) | 0.445 |
| Tumor size (**≥** 5 vs < 5 cm) | 1.090 (0.257-4.615) | 0.907 |  | 0.997 (0.205-4.842) | 0.997 |
| Tumor invasion (ACC vs ECC) | 1.023 (0.310-3.369) | 0.971 |  | 1.579 (0.421-5.921) | 0.498 |
| LN metastasis (positive vs negative) | 3.549 (1.540-8.181) | 0.003 |  | 0.081 (0.009-0.730) | 0.025 |
| Vessel invasion (positive vs negative) | 2.004 (0.989-4.059) | 0.054 |  | 0.618 (0.300-1.273) | 0.191 |
| Distant metastasis (positive vs negative) | 1.859 (0.712-4.856) | 0.206 |  | 0.089 (0.009-0.901) | 0.041 |
| TNM stage (stage III and IV vs I and II) | 6.296 (1.916-20.688) | 0.002 |  | 1.093 (0.167-7.144) | 0.926 |
| Tissue DC-SIGN (high vs low) | 0.160 (0.034-0.752) | 0.021 |  |  |  |
| Serum DC-SIGN (high vs low) | 2.121 (0.955-4.711) | 0.065 |  | 1.179 (0.459-3.028) | 0.732 |

**Abbreviations:** OS, overall survival; HR, hazard ratio; CI, conﬁdence interval; LN, lymph node; ECC, early colorectal cancer; ACC, advanced colorectal cancer.

**Supplementary Table S3.** *CD209* Mutation in The Cancer Genome Atlas Database

| **Sample ID** | **Mutation** | **Coding DNA Change** | **Mutation assessor^a^** | **SIFT^b^** | **Polymorphism phenotyping^c^** |
| --- | --- | --- | --- | --- | --- |
| TCGA-AA-3812-01 | G55E | 164G>A | Medium | Deleterious | Benign |
| TCGA-AA-3977-01 | E93D | 279G>T | Medium | Tolerant | Probably damaging |
| TCGA-AG-A02N-01 | A283T | 847G>A | Neutral | Deleterious | Probably damaging |

^a^Predicted functional impact score (via Mutation Assessor) for missense mutations.

^b^Predicted whether an amino acid substitution affects protein function (via SIFT) for missense mutations.

^c^Predicted possible functional effects of human nsSNPs (via PolyPhen-2) for missense mutations.

**Supplementary Table S4.** At least 3 databases of miRNAs binding sites in human DC-SIGN 3’-UTR.

| **miRNAs** | **Mature sequence** | **Location** | **Site** | **Free Energy (kcal/mol)** | **Database** | | | | |
| --- | --- | --- | --- | --- | --- | --- | --- | --- | --- |
|  |  |  |  |  | **TargetScan** | **miRanda** | **RNA22** | **miRDB** | **TarBase** |
| miR-185-5p | TGGAGAGAAAGGCAGTTCCTGA | 34-41 | 8mer | -24.5 | ***** | ***** | ***** | ***** | ***** |
| miR-485-5p | AGAGGCTGGCCGTGATGAATTC | 681-688 | 8mer | -25.2 | ***** | ***** | ***** |  |  |
| miR-326 | CCTCTGGGCCCTTCCTCCAG | 454-461 | 8mer | -25.0 | ***** |  | ***** | ***** |  |
| miR-6780b-5p | TGGGGAAGGCTTGGCAGGGAAGA | 2398-2405 | 8mer | -32.5 | ***** |  | ***** | ***** |  |
| miR-4486 | GCTGGGCGAGGCTGGCA | 1708-1714 | 7mer-m8 | -28.7 | ***** |  | ***** | ***** |  |
| miR-4534 | GGATGGAGGAGGGGTCT | 37-43 | 7mer-m8 | -28.1 | ***** |  | ***** | ***** |  |
| miR-4722-5p | GGCAGGAGGGCTGTGCCAGGTTG | 357-364 | 8mer | -26.4 | ***** |  | ***** | ***** |  |
| miR-486-3p | CGGGGCAGCTCAGTACAGGA | 2955-2962 | 8mer | -26.0 | ***** |  | ***** | ***** |  |
| miR-4664-5p | TGGGGTGCCCACTCCGCAAGTT | 362-368 | 7mer-m8 | -23.6 | ***** |  | ***** | ***** |  |
| miR-4316 | GGTGAGGCTAGCTGGTG | 2929-2935 | 7mer-m8 | -21.4 | ***** |  | ***** | ***** |  |
| miR-2392 | TAGGATGGGGGTGAGAGGTG | 39-45 | 7mer-m8 | -25.3 | ***** |  | ***** | ***** |  |
| miR-765 | TGGAGGAGAAGGAAGGTGATG | 941-947 | 7mer-m8 | -26.7 | ***** |  | ***** | ***** |  |
| miR-6802-5p | CTAGGTGGGGGGCTTGAAGC | 346-352 | 7mer-m8 | -23.5 | ***** |  | ***** | ***** |  |
| miR-6849-3p | ACCAGCCTGTGTCCACCTCCAG | 101-107 | 7mer-m8 | -22.7 | ***** |  | ***** | ***** |  |
| miR-608 | AGGGGTGGTGTTGGGACAGCTCCGT | 489-495 | 7mer-m8 | -32.4 | ***** |  | ***** | ***** |  |
| miR-449b-3p | CAGCCACAACTACCCTGCCACT | 2537-2543 | 7mer-m8 | -25.3 | ***** |  | ***** | ***** |  |
| miR-1270 | CTGGAGATATGGAAGAGCTGTGT | 2045-2052 | 8mer | -22.3 | ***** |  | ***** | ***** |  |
| miR-4303 | TTCTGAGCTGAGGACAG | 2789-2795 | 7mer-m8 | -21.0 | ***** |  | ***** | ***** |  |
| miR-4478 | GAGGCTGAGCTGAGGAG | 2170-2176 | 7mer-m8 | -24.1 | ***** |  | ***** | ***** |  |
| miR-571 | TGAGTTGGCCATCTGAGTGAG | 2159-2166 | 8mer | -22.2 | ***** |  | ***** | ***** |  |
| miR-342-5p | AGGGGTGCTATCTGTGATTGA | 362-368 | 7mer-m8 | -23.7 | ***** |  | ***** | ***** |  |
| miR-4656 | TGGGCTGAGGGCAGGAGGCCTGT | 2653-2660 | 8mer | -32.2 | ***** |  | ***** | ***** |  |
| miR-1587 | TTGGGCTGGGCTGGGTTGGG | 2654-2661 | 8mer | -30.6 | ***** |  | ***** | ***** |  |
| miR-6134 | TGAGGTGGTAGGATGTAGA | 2309-2315 | 7mer-m8 | -21.8 | ***** |  | ***** | ***** |  |
| miR-6734-5p | TTGAGGGGAGAATGAGGTGGAGA | 2900-2906 | 7mer-m8 | -29.5 | ***** |  | ***** | ***** |  |
| miR-5088-3p | TCCCTTCTTCCTGGGCCCTCA | 2004-2010 | 7mer-m8 | -26.2 | ***** |  | ***** | ***** |  |
| miR-671-5p | AGGAAGCCCTGGAGGGGCTGGAG | 520-527 | 8mer | -30.9 | ***** |  | ***** | ***** |  |
| miR-4306 | TGGAGAGAAAGGCAGTA | 34-41 | 8mer | -21.3 | ***** |  | ***** | ***** |  |
| miR-6077 | GGGAAGAGCTGTACGGCCTTC | 246-252 | 7mer-m8 | -23.4 | ***** |  | ***** | ***** |  |
| miR-4640-5p | TGGGCCAGGGAGCAGCTGGTGGG | 166-173 | 8mer | -34.3 | ***** |  | ***** | ***** |  |
| miR-6763-5p | CTGGGGAGTGGCTGGGGAG | 1513-1519 | 7mer-m8 | -25.7 | ***** |  | ***** | ***** |  |
| miR-4267 | TCCAGCTCGGTGGCAC | 193-199 | 7mer-m8 | -21.5 | ***** |  | ***** | ***** |  |
| miR-4726-5p | AGGGCCAGAGGAGCCTGGAGTG | 166-173 | 8mer | -33.3 | ***** |  | ***** | ***** |  |
| miR-4443 | TTGGAGGCGTGGGTTTT | 132-139 | 8mer | -21.3 | ***** |  | ***** | ***** |  |
| miR-646 | AAGCAGCTGCCTCTGAGGC | 287-294 | 8mer | -23.5 | ***** |  | ***** | ***** |  |
| miR-6770-5p | TGAGAAGGCACAGCTTGCACGTGA | 31-37 | 7mer-m8 | -24.3 | ***** |  | ***** | ***** |  |
| miR-3184-5p | TGAGGGGCCTCAGACCGAGCTTTT | 1464-1471 | 8mer | -27.2 | ***** |  | ***** | ***** |  |
| miR-3620-5p | GTGGGCTGGGCTGGGCTGGGCC | 2654-2661 | 8mer | -35.7 | ***** |  | ***** | ***** |  |
| miR-4688 | TAGGGGCAGCAGAGGACCTGGG | 1463-1469 | 7mer-m8 | -27.8 | ***** |  | ***** | ***** |  |
| miR-6847-5p | ACAGAGGACAGTGGAGTGTGAGC | 2249-2255 | 7mer-m8 | -25.4 | ***** |  | ***** | ***** |  |
| miR-4447 | GGTGGGGGCTGTTGTTT | 492-499 | 8mer | -23.0 | ***** |  | ***** | ***** |  |
| miR-4651 | CGGGGTGGGTGAGGTCGGGC | 489-495 | 7mer-m8 | -27.1 | ***** |  | ***** | ***** |  |
| miR-3929 | GAGGCTGATGTGAGTAGACCACT | 2170-2176 | 7mer-m8 | -27.5 | ***** |  | ***** | ***** |  |
| miR-3150a-3p | CTGGGGAGATCCTCGAGGTTGG | 1513-1519 | 7mer-m8 | -31.2 | ***** |  | ***** | ***** |  |
| miR-423-5p | TGAGGGGCAGAGAGCGAGACTTT | 2600-2606 | 7mer-m8 | -26.3 | ***** |  |  | ***** | * |

**Abbreviations:** 8mer, an exact match to positions 2-8 of the mature sequence (5' to 3') followed by an 'A'; 7mer-m8, an exact match to positions 2-8 of the mature sequence (5' to 3'); 7mer-A1, an exact match to positions 2-7 of the mature sequence (5' to 3') followed by an 'A'; non, non-canonical sites.

**Supplementary Table S5.** Potential LEF1/TCF1 binding site on miR-185 promoter.

| **Gene** | **Score** | **Start** | **End** | **Strand** | **Predicted site sequence** |
| --- | --- | --- | --- | --- | --- |
| TCF1 | 8.792 | 64 | 77 | - | GGCTAATTTTTTTT |
| TCF1 | 6.01615 | 684 | 697 | - | TGTTCATTTGTAGC |
| LEF1 | 4.69085 | 913 | 927 | + | TGAGATCACAGCTGC |
| TCF1 | 7.86215 | 1117 | 1130 | - | GGAAAATGCTTACC |
| LEF1 | 6.80489 | 1412 | 1426 | - | AGTGGTCAAAGCCAT |
| LEF1 | 3.14702 | 1792 | 1806 | + | AGGCATGAGAGGGTG |

**Supplementary Table S6.** The antibodies used in this study.

| **Name** | **Host** | **Product code** | **Company** |
| --- | --- | --- | --- |
| DC-SIGN (for WB, IHC) | Rabbit pAb | ab5715 | Abcam |
| DC-SIGN (for ELISA) | Mouse mAb | SAB1403221 | Sigma-Aldrich |
| DC-SIGN-APC (for IF) | rat monoclonal | 17-2099-42 | eBioscience |
| DC-SIGN-APC (for Flow Cyt) | Mouse mAb | 330108 | BioLegend |
| CEA | Rabbit pAb | BS6073 | Bioworld |
| CD11c | Mouse mAb | 60258-1-Ig | Proteintech |
| MMP-9 | Rabbit mAb | ZA-0562 | ZSGB-BIO |
| VEGF | Rabbit pAb | BA0407 | BOSTER |
| Lyn (for WB) | Mouse mAb | 4576 | Cell Signaling |
| Lyn (for IP) | Rabbit mAb | 2796 | Cell Signaling |
| phospho-Akt | Mouse mAb | sc-271966 | Santa Cruz |
| Akt | Mouse mAb | sc-5298 | Santa Cruz |
| phospho-GSK-3β | Mouse mAb | sc-373800 | Santa Cruz |
| GSK-3β | Mouse mAb | sc-377213 | Santa Cruz |
| β-catenin (for WB) | Mouse mAb | sc-7963 | Santa Cruz |
| β-catenin (for IF) | Rabbit pAb | [51067-2-AP](http://www.ptgcn.com/products/b-cat-Antibody-51067-2-AP.htm) | Proteintech |
| LEF-1 (for WB, IHC) | Mouse mAb | sc-374412 | Santa Cruz |
| LEF-1 (for ChIP) | Rabbit mAb | 76010 | Cell Signaling |
| TCF-1 (for WB, IHC) | Mouse mAb | sc-393925 | Santa Cruz |
| TCF-1 (for ChIP) | Mouse mAb | sc-393925X | Santa Cruz |
| phosphotyrosine | Mouse mAb | ab10321 | Abcam |
| CK20 | Rabbit pAb | BS6026 | Bioworld |
| p85 | Mouse mAb | sc-376112 | Santa Cruz |
| P110 | Mouse mAb | sc-8010 | Santa Cruz |
| Ki-67 | Rabbit pAb | BS6667 | Bioworld |
| PCNA | Mouse mAb | sc-56 | Santa Cruz |
| CK7 | Rabbit pAb | 17513-1-AP | Proteintech |
| CDX2 | Rabbit mAb | ab76541 | Abcam |
| phospho-ERK1/2 | Mouse mAb | sc-7383 | Santa Cruz |
| ERK1/2 | Mouse mAb | sc-514302 | Santa Cruz |
| β-actin | Mouse mAb | TA-09 | ZSGB-BIO |
| Myc-Tag | Rabbit mAb | 2278 | Cell Signaling |
| FLAG-Tag | Rabbit pAb | 2368 | Cell Signaling |

**Supplementary Table S7.** The names of the genes and sequences of primers.

| **Gene name** | **Sequence (5' to 3')** | | **Amplified size** |
| --- | --- | --- | --- |
|  | **Sence** | **Anti-sence** |  |
| **Normal PCR Primers** | | | |
| *CD209* | ATTTTCCAACTCATTTTCAGCC | TCTCACAGAAAGAGGAGGACAC | 305 |
| *β-actin* | CGAAAGTTGCCTTTTATGGCTC | GCCATCTCTTGCTCGAAGTC | 838 |
| **Quantitative RT-PCR Primers** | | | |
| *CD209* | GAAGTAACCGCTTCACCTGGAT | AATTCCGCGCAGTCTTCCT | 151 |
| *Lyn* | TGTGAGAGATCCAACGTCCA | GAAAGACAAGTCGTCCGGGT | 157 |
| *MMP-9* | CCCCTTCACTTTCCTGGGTAA | CGCCACGAGGAACAAACTGT | 151 |
| *MMP-7* | AACTCCCGCGTCATAGAAAT | GATACGATCCTGTAGGTGAC | 122 |
| *MMP-2* | AAGTATGGCTTCTGCCCTGA | ATTTGTTGCCCAGGAAAGTG | 97 |
| *VEGF* | GCAGAAGGAGGAGGGCAGAATC | ACACTCCAGGCCCTCGTCATT | 149 |
| *MET* | CAGATGTGTGGTCCTTTG | ATTCGGGTTGTAGGAGTCT | 129 |
| *ITGB1* | GCCTGTGGAGTACAAGTCCTT | AATTCGGGTGAAGTTATCTGTGG | 163 |
| *CDH1* | GTCAGGTGCCTGAGAACGAG | GCCATCGTTGTTCACTGGAT | 158 |
| *DRG1* | AGGCGGACATTCTGGAAATG | CGGTACTTCCCCAGCACACTT | 103 |
| *SMAD7* | TGCTCCCATCCTGTGTGTTAAG | TCAGCCTAGGATGGTACCTTGG | 135 |
| *LEF1* | AGAACACCCCGATGACGGA | GGCATCATTATGTACCCGGAAT | 90 |
| *TCF1* | AGATCCTGTTCCAGGCCTAT | GGATGCATTCCGCCCTATT | 93 |
| *TCF2* | TGTTGCATGTATCCCTTG | TCAGCCTAGGATGGTACCTTGG | 144 |
| *TCF4* | GGCTATGCAGGAATGTTGGG | GTTCATGTGGATGCAGGCTAC | 76 |
| *SRY* | AGAGAATCCCAGAATGCGAAAC | CTTCCGACGAGGTCGATACTT | 163 |
| *SP1* | TCCAGACCATTAACCTCAGTGC | TGTATTCCATCACCACCAGCC | 142 |
| *PAX5* | AAACCAAAGGTCGCCACAC | GTTGATGGAACTGACGCTAGG | 150 |
| *ETS1* | CTAGCTGGGTGAAACCCTTATT | CCAGAATGGAGAAGGGAACAA | 98 |
| *NFIC* | ACCTGGCATACGACCTGAAC | TCCATCGAGCCCGATTTGTG | 100 |
| *P65* | AGCTCAAGATCTGCCGAGTG | ACATCAGCTTGCGAAAAGGA | 154 |
| *E2F1* | CATCAGTACCTGGCCGAGAG | TGGTGGTCAGATTCAGTGAGG | 118 |
| *NR2F1* | CTTCGTCCGTTTGGTAGGTAAA | GAGCACTGGATGGACATGTAAG | 102 |
| *FOXP3* | CACTCACCTCACTCCCATTC | GGGCCTTGGATCCCAAATAA | 89 |
| *P85* | TGGACGGCGAAGTAAAGCATT | AGTGTGACATTGAGGGAGTCG | 154 |
| *P110* | AGTAGGCAACCGTGAAGAAAAG | GAGGTGAATTGAGGTCCCTAAGA | 167 |
| *PTEN* | TGAGTTCCCTCAGCCGTTACCT | GAGGTTTCCTCTGGTCCTGGTA | 138 |
| *GAPDH* | GTCAAGGCTGAGAACGGGAA | AAATGAGCCCCAGCCTTCTC | 158 |
| *U6* | CTCGCTTCGGCAGCACA | AACGCTTCACGAATTTGCGT |  |
| **Mutation Primers** | | | |
| *CD209 G55E* | CACGCTCTTGGCTGAGCTCCTTG  TCCAAGT | AAGGAGAGGAGTTGCAGCACCA  GGGGACCA |  |
| *CD209 E93D* | GGTGAGCTCTCAGATAAATCCAA  GCTGCAG | CACTGCAGCTTTGAGCTGGGTCA  GG |  |
| *CD209 A283T* | ACGACTCCATCACCACCTGCAAA  GAAGTGG | GCCAGTTCCGCTGGGAGTTAGAC  ATGAAGT |  |
| *CD209*  *LL*–*AA* | AGCAGCTGGGCGCCGCGGAGGA  GGAACA | GCAGTCTTGGTTCCTTGGAGTCA  CTCAT |  |
| *CD209 Y31A* | AGACTCGAGGAGCCAAGAGCTTA  GCAG | GTCGGAATCCAAGGCCTCTCAGC  TG |  |
| **ChIP Primers** | | | |
| *S1* | TGGAAGTTGTGGGAAGTGGTC | CTAGGTCCTACAAGGTCCCCA | 185 |
| *S2* | TCCTGCAGATGTTCAGATGCT | CAAGAAGGCCAGAGTGGTCAA | 176 |
| *S3* | ACCTACTTGCCCAACTGCTA | CCAAGGGGCTAAGACCACC | 161 |
| *S4* | CTGCTTCTTCACCTCTAGCCC | CACAGGTGGGAAGATGCTGAT | 160 |
| *Ctrl S* | GTCGCTTGATGGGACGAAGA | TGTGGGGTTAACACGCTAGG | 158 |
